# Supplementary material for: Pervasive tandem duplications and convergent evolution shape coral genomes
Source: Genome Biol. 2023 Jun 1;24:123. doi: 10.1186/s13059-023-02960-7 (PMC10236652; doi:10.1186/s13059-023-02960-7)

Table S1: Statistics of the ONT sequencing data

	Porites lobata Assembly size of 650Mb			*Pocillopora* cf. *effusa* Assembly size of 350Mb		
	Raw ONT reads	Longest reads	Filtlong reads	Raw ONT reads	Longest reads	Filtlong reads
Number of runs	4 MinION + 4 PromethION			4 MinION + 1 PromethION		
Cumulative size	156 Gb	21.0 Gb	21.0 Gb	62 Gb	10.5 Gb	10.5 Gb
# of reads	55,129,048	585,173	770,592	13,260,136	191,244	286,789
Coverage	240X	32X	32X	177X	30X	30X
N50 (bp)	5,189	35,377	28,893	15,605	53,865	40,276
Coverage (>50Kb)	6X	6X	4X	18X	18X	10X

	Porites evermanni Assembly size of 650Mb				
	Paired-end reads	Mate pairs			
Sequencer	HiSeq 2500	HiSeq 4000	HiSeq 4000	HiSeq 4000	HiSeq 4000
Cumulative size	117.6 Gb	23.1 Gb	19.1 Gb	10.6 Gb	13.7 Gb
# of read pairs	235,175,203	94,673,617	77,765,666	43,308,999	56,684,886
Coverage	181X	35X	29X	16X	21X
Read size (bp)	2x250	2x150	2x150	2x150	2x150
Insert size (bp)	400	4,000	7,000	9,000	14,000

Table S2: Raw long-read assemblies. A. *Porites lobata*. **B.** *Pocillopora* cf. *effusa*.

**A**	SMARTdenovo			Flye		
Subset of reads used	All reads	Filtlong	Longest	All reads	Filtlong	Longest
# contigs	2,225	2,149	2,098	9,845	6,771	6,287
Cumulative size	841,373,280	776,733,905	754,545,205	865,703,755	870,048,965	920,835,732
N50 (L50)	752,073 (334)	677,273 (332)	671,074 (311)	274,490 (906)	221,134 (1,143)	251,501 (1,014)
N90 (L90)	171,297 (1,237)	144,573 (1,285)	135,601 (1,287)	54,588 (3,657)	72,000 (3,837)	79,349 (3,575)
Max size	4,191,972	3,275,313	4,259,348	2,045,142	1,531,077	1,741,775
	Ra			RedBean		
Subset of reads used	All reads	Filtlong	Longest	All reads	Filtlong	Longest
# contigs	-	2,992	2,693	30,253	5,792	5,429
Cumulative size	-	374,907,362	433,765,815	761,471,974	668,429,293	692,777,752
N50 (L50)	-	151,282 (792)	194,050 (681)	47,890 (3,593)	383,820 (436)	393,812 (455)
N90 (L90)	-	68,872 (2,238)	85,574 (2,032)	9,095 (18,619)	51,918 (2,298)	62,171 (2,154)
Max size	-	897,817	1,051,442	810,039	2,685,953	2,863,807

**B**	SMARTdenovo			Flye		
Subset of reads used	All reads	Filtlong	Longest	All reads	Filtlong	Longest
# contigs	388	410	405	2,725	2,287	2,421
Cumulative size	371,884,384	356,631,128	343,867,399	399,792,282	381,694,599	407,450,451
N50 (L50)	2,617,398 (44)	2,617,493 (45)	2,511,303 (44)	368,823 (282)	372,078 (256)	318,427 (340)
N90 (L90)	380,661 (182)	336,700 (173)	293,979 (185)	84,125 (1,216)	84,025 (1,221)	90,251 (1,300)
Max size	8,947,962	7,928,004	6,858,622	2,561,294	3,845,024	2,697,919
	Ra			RedBean		
Subset of reads used	All reads	Filtlong	Longest	All reads	Filtlong	Longest
# contigs	-	-	-	12,023	1,185	1,300
Cumulative size	-	-	-	382,648,274	312,572,668	324,887,521
N50 (L50)	-	-	-	67,474 (1,118)	2,012,258 (41)	1,541,157 (60)
N90 (L90)	-	-	-	11,305 (6,805)	134,012 (259)	133,570 (303)
Max size	-	-	-	1,902,040	10,295,455	8,452,070

Table S3: Statistics of all assemblies. The two columns for Haplomerger2 correspond to the two haplotypes generated by the software (a reference haplotype and an alternative haplotype). Two-copy k-mers correspond to duplicated contigs or overlaps in the assembly.

	Porites lobata			
Method	Raw assembly	Purge Dups	Haplomerger2 Reference hap	Haplomerger2 Alternative hap
# contigs	2,225	1,057	1,098	1,158
Cumulative size	851,308,987	587,952,820	646,152,978	588,081,904
N50 (L50)	763,922 (333)	977,395 (189)	2,154,615 (84)	1,959,703 (84)
N90 (L90)	173,088 (1,236)	302,768 (587)	302,418 (357)	253,346 (374)
Max size	4,260,399	4,260,399	8,615,247	7,759,842
BUSCO eukaryota odb10 (N=255)	C:98.4% D:25.1% F:0.4% M:1.2%	C:97.7% D:2.0% F:1.2% M:1.1%	C:98.9% D:2.4% F:0.0% M:1.1%	C:98.5% D:2.0% F:0.4% M:1.1%
Number of two-copy k-mers	77,298,864	26,805,171	31,535,458	28,078,888

	Pocillopora cf. *effusa*			
Method	Raw assembly	Purge Dups	Haplomerger2 Reference hap	Haplomerger2 Alternative hap
# contigs	388	388	252	255
Cumulative size	376,159,770	376,159,770	347,233,126	340,785,530
N50 (L50)	2,645,240 (44)	2,645,240 (44)	4,753,879 (23)	4,593,186 (23)
N90 (L90)	392,528 (181)	392,528 (181)	955,142 (81)	905,508 (81)
Max size	9,057,404	9,057,404	11,895,822	11,581,782
BUSCO eukaryota odb10 (N=255)	C:98.8% D:7.8% F:0.4% M:0.8%	C:98.8% D:7.8% F:0.4% M:0.8%	C:98.9% D:2.4% F:0.4% M:0.7%	C:98.8% D:2.7% F:0.4% M:0.8%
Number of two-copy k-mers	25,406,945	25,406,945	17,008,283	16,631,927

Table S4: Statistics of gene prediction on reference and alternative haplotype assemblies.

	Porites lobata		***Pocillopora* cf. *effusa***	
	Reference haplotype	*Alternative haplotype*	*Reference haplotype*	*Alternative haplotype*
number of genes	42,872	45,845	32,095	34,311
number of single-exon genes	7,496	10,906	5,341	7,135
Average CDS size (bp)	1,384	1,166	1,458	1,284
Average number of exons per gene	5.68	4.89	6.53	5.79
BUSCO scores (N=954)	C:95.0% [S:91.3%,D:3.7%] F:1.8% M:3.2%	C:88.7% [S:85.1%,D:3.6%] F:5.8% M:5.5%	C:94.8% [S:92.0%,D:2.8%] F:2.4% M:2.8%	C:91.6% [S:88.6%,D:3.0%] F:4.7% M:3.7%

Table S5: BUSCO analyses of raw and haploid assemblies.

	Porites lobata			*Pocillopora* cf. *effusa*		
metazoa_odb10 N=954 genes	Raw assembly	Polished assembly	Haploid assembly	Raw assembly	Polished assembly	Haploid assembly
Complete	92.2%	95.9%	95.8%	89.9%	95.8%	95.9%
Duplicated	17.8%	26.6%	3.7%	3.1%	6.2%	2.8%
Fragmented	2.9%	1.6%	1.5%	4.7%	1.9%	1.8%
Missing	4.9%	2.5%	2.7%	5.4%	2.3%	2.3%

Table S6: Repeat composition of coral assemblies.

	Porites lobata	*Pocillopora* cf. *effusa*
bases masked	56.24 %	40.30 %
Retroelements	10.02 %	10.50 %
DNA transposons	17.40 %	9.00 %
Unknown TE	20.19 %	13.17 %
Simple repeats	5.83 %	4.63 %
Low complexity	0.68 %	1.34 %
Satellite	0.27 %	0.07 %
Rolling-circles	1.54 %	0.91 %
Small RNA	0.30 %	0.67 %

Table S7: Data used for comparison of 26 Cnidaria species. Accession numbers of short-reads datasets used to estimate genome size and abundance of the different OGs in each species, and links to assemblies and annotations downloaded.

Species	Run accessions (Illumina short-reads)	BioProject accessions (assemblies)	Genome annotations	References
Acropora digitifera	DRR194164		http://spis.reefgenomics.org/download/	Shinzato, 2011
Acropora millepora v1.1 *(-> comparative analyses: Figure 1E,F)*	SRR8119879, SRR8119880	PRJNA473876	http://coralreefgenomes.jcu.edu.au/	Ying, 2019
v2.1 (-> assembly metrics: Figure 1 B,C,D,G)	-	PRJNA633778	https://ftp.ncbi.nlm.nih.gov/genomes/all/GCF/013/753/865/GCF_013753865.1_Amil_v2.1/GCF_013753865.1_Amil_v2.1_translated_cds.faa.gz	Fuller,2020
Aiptasia sp	SRR1648348		http://aiptasia.reefgenomics.org/	Baumgarten, 2015
Amplexidiscus fenestrafer	SRR5123099, SRR5123102, SRR5123104	PRJNA354436	http://corallimorpharia.reefgenomics.org/	Wang, 2016
Aurelia aurita (Atlantic)	-	PRJNA494057	https://marinegenomics.oist.jp/aurelia_aurita	Khalturin, 2019
Aurelia aurita (Pacific)	-	PRJNA494062	https://marinegenomics.oist.jp/aurelia_aurita_pacific	Khalturin, 2019
Clytia hemisphaerica	-	PRJEB30490	http://marimba.obs-vlfr.fr/	Leclère, 2019
Dendronephtya gigantea	SRR8293698	PRJNA507923	GCF_004324835.1	Jeon, 2019
Discosoma sp	SRR5131523, SRR5131524, SRR5131536	PRJNA354492	http://corallimorpharia.reefgenomics.org/	Wang, 2016
Fungia spp.	ERR2190368, ERR2190369	PRJEB23312	http://ffun.reefgenomics.org/	Ying, 2018
Galaxea fascicularis	ERR2191366	PRJEB23333	http://gfas.reefgenomics.org/	Ying, 2018
Goniastrea aspera	ERR2192485, ERR2192486	PRJEB23371	http://gasp.reefgenomics.org/	Ying, 2018
Hydra vulgaris	-		https://research.nhgri.nih.gov/hydra/	
Montipora capitata	SRR8497577	PRJNA509219	http://cyanophora.rutgers.edu/montipora/	Shumaker, 2019
Morbakka virulenta	-	PRJNA494059	https://marinegenomics.oist.jp/morbakka_virulenta/	Khalturin, 2019
Nematostella vectensis	-	GCA_000209225.1	ftp://ftp.ensemblgenomes.org/pub/metazoa/release-43/fasta/nematostella_vectensis	Putnam, 2007
Orbicella faveolata	SRR4842281	PRJNA342412	ftp://ftp.ncbi.nlm.nih.gov/genomes/Orbicella_faveolata	Prada, 2016
Pocillopora acuta	SRR4254617		http://ihpe.univ-perp.fr/telechargement/Data_to_download.rar	Vidal-Dupiol, 2019
Pocillopora damicornis	-	PRJNA454489	http://pdam.reefgenomics.org/	Cunning, 2018
Pocillopora* cf. *effusa	**ERR9321698**	**PRJEB51538**	**http://www.genoscope.cns.fr/corals**	**this study**
Pocillopora verrucosa	SRR11880677		http://pver.reefgenomics.org/	Buitrago-López, 2020
Porites evermani	**ERR9356150, ERR9356151**	**PRJEB51771**	**http://www.genoscope.cns.fr/corals**	**this study**
Porites lobata	**ERR9321731**	**PRJEB51842**	**http://www.genoscope.cns.fr/corals**	**this study**
Porites lutea	ERR571457, ERR571458, ERR571459	PRJEB6884	http://plut.reefgenomics.org/	Robbins, 2019
Porites rus	-	PRJEB23570	http://gigadb.org/dataset/100462	Celis, 2018
Stylophora pistillata	SRR1980957, SRR1980958	PRJNA281535	http://spis.reefgenomics.org/	Voolstra, 2017

Table S8: Coverage of genome assemblies by syntenic blocks (for contigs with at least 5 genes).

	Porites lobata	*Porites lutea*	*Pocillopora* cf. *effusa*	*Pocillopora verrucosa*
Porites lobata	-	397 Mb (78%)	260 Mb (75%)	136 Mb (45%)
Porites lutea	457 Mb (73%)	-	239 Mb (69%)	117 Mb (39%)
Pocillopora cf. *effusa*	439 Mb (70%)	305 Mb (60%)	-	186 Mb (61%)
Pocillopora verrucosa	330 Mb (53%)	216 Mb (42%)	210 Mb (61%)	-

Table S9: Validation of pairs of adjacent duplicate genes and clusters of tandemly duplicated genes. Two adjacent genes are considered validated if at least one ONT read completely overlaps the two genes. Similarly, a cluster of TDG is considered validated if at least one ONT read completely overlaps all genes of the cluster (Figure S6).

		Porites lobata		*Pocillopora* cf. *effusa*	
		Validated	Not validated	Validated	Not validated
Pairs of adjacent duplicated genes	Number	5,780 (70.3%)	2,440 (29.7%)	5,973 (91.2%)	579 (8.8%)
	Average size of the genomic region (bp)	23,594	89,790	23,865	95,434
	Median size of the genomic region (bp)	20,781	79,530	19,990	86,723
Clusters of TDG	Number	1,680 (45.5%)	2,012 (54.5%)	1,838 (67.0%)	903 (33.0%)
	Average size of the genomic region (bp)	27,316	144,391	31,700	150,589
	Median size of the genomic region (bp)	24,990	109,211	27,771	115,593

Table S10: Table of calcification-related proteins in non-scleractinian and scleractinian - robust and - complex coral species.

Hexacorallia	Amt1	SLC4		PMCA	CA	CARP	Neurexin
		β	ɣ				
**Corallimorpharia**							
Discosoma sp.	1	1	0	2	6	2	4
Amplexidiscus fenestrafer	1	1	0	2	6	2	4
**Actinaria**							
Exaiptasia diaphana	2	1	0	3	10	2	3
**Scleractinia - "Robust"**							
Stylophora pistillata	5	1	1	3	16	5	3
Pocillopora verrucosa	4	1	1	3	14	5	4
Pocillopora cf. *effusa*	5	1	1	3	15	5	3
Orbicella faveolata	4	1	1	3	14	4	4
Goniastrea aspera	4	1	1	3	12	4	3
Fungia sp.	2	1	1	3	14	4	3
**Scleractinia - "Complex"**							
Acropora digitifera	2	1	1	3	10	3	3
Acropora millepora	2	1	1	3	10	3	3
Galaxea fascicularis	1	1	1	3	9	3	4
Montipora capitata	2	1	1	3	9	5	3
Porites lobata	3	1	1	3	10	5	5
Porites lutea	3	1	1	1	11	3	4

Figure S1. K-mer distribution and estimation of genome sizes and heterozygous rates from short reads. (A) *Porites lobata*, (B) *Porites evermanni*, and (C) *Pocillopora* cf. *effusa.*

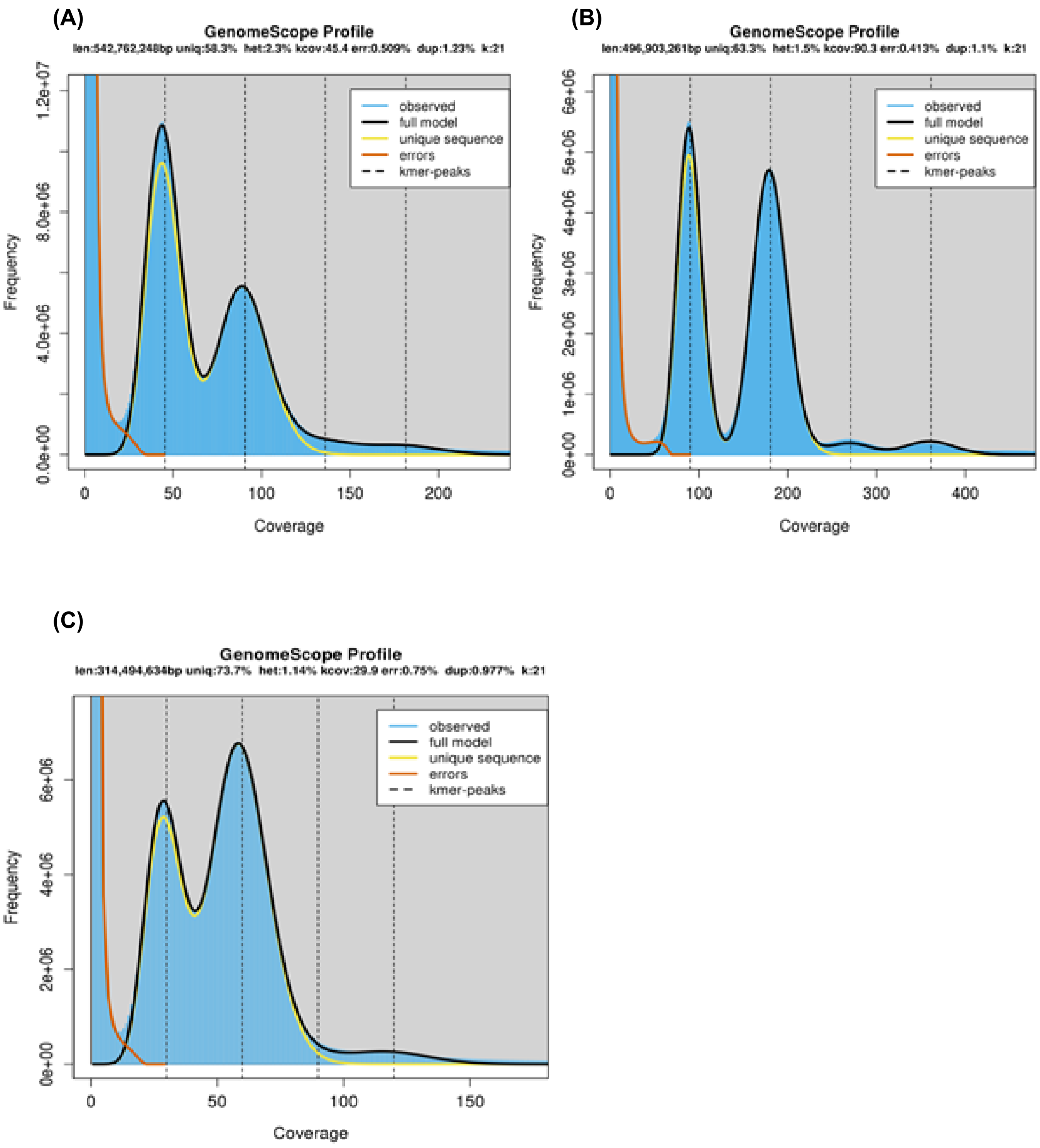

Figure S2. KAT plot of *Porites lobata* assemblies. (A) polished nanopore assembly. (B) haploid version of the genome.

A

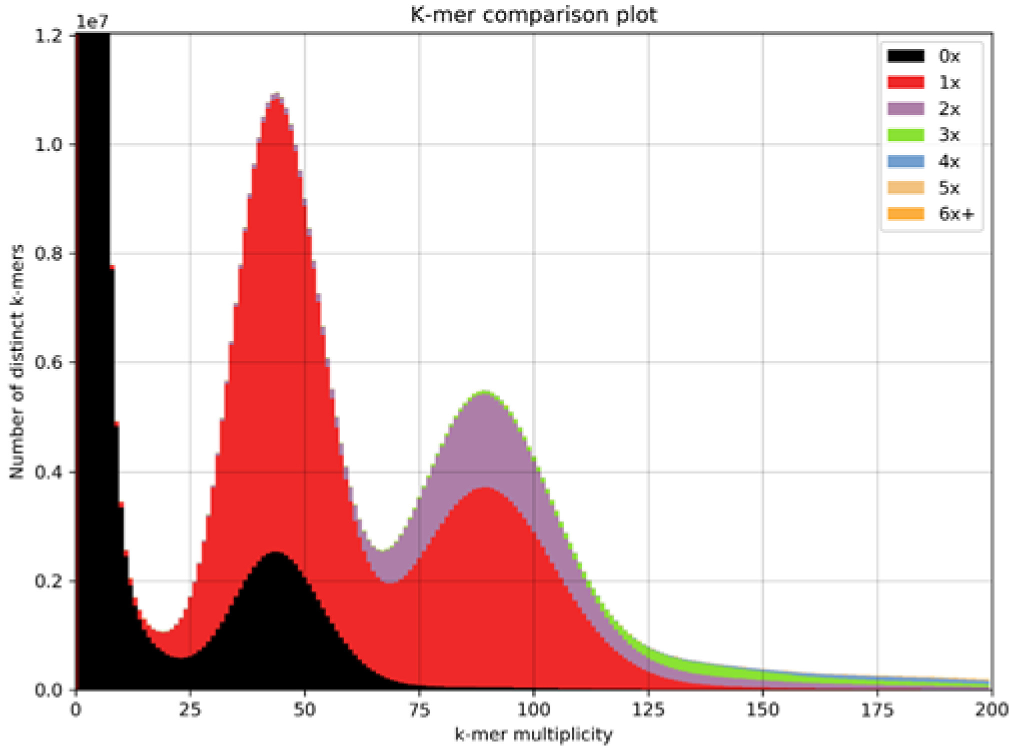

B

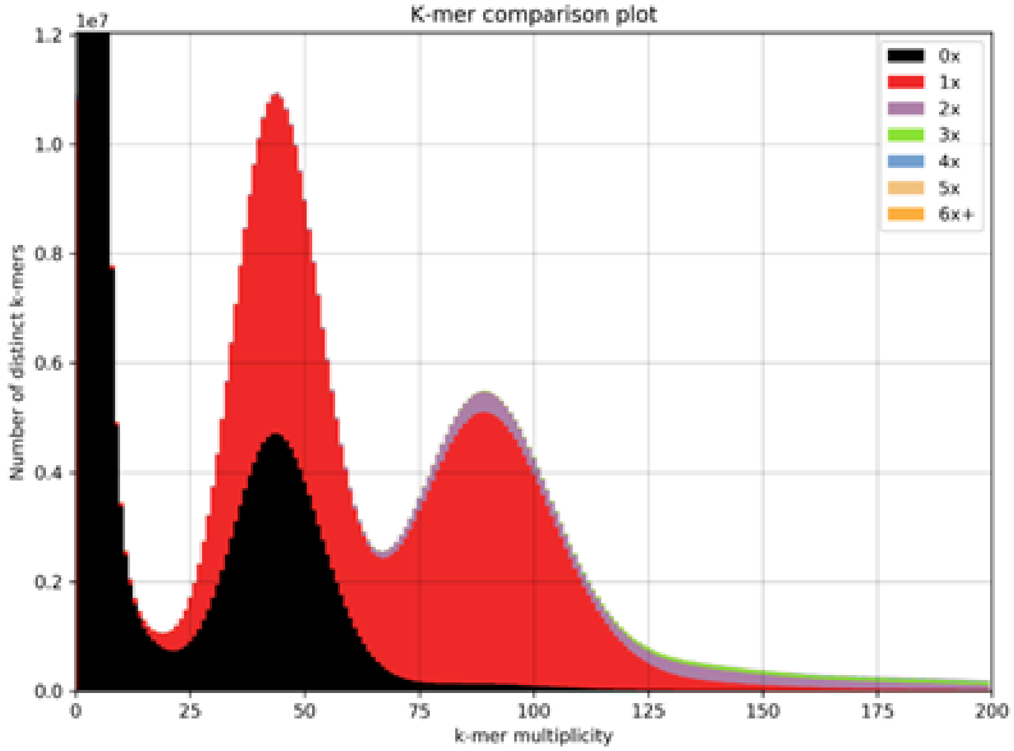

Figure S3. KAT plot of *Pocillopora* cf. *effusa* assemblies. (A) polished nanopore assembly. (B) haploid version of the genome.

A

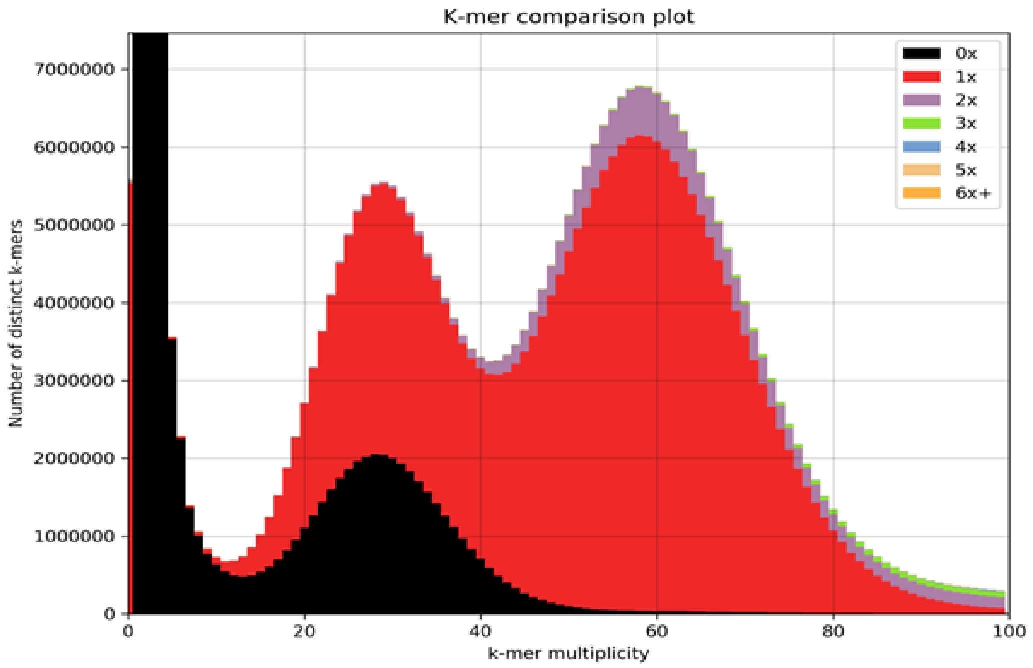

B

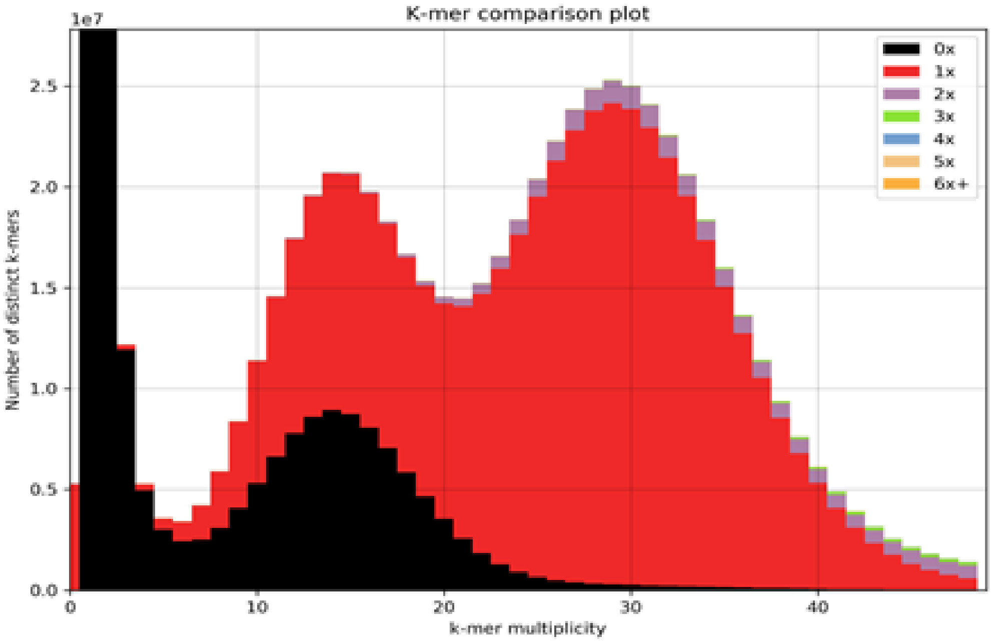

Figure S4. K-mer profiling for all assemblies of *Porites lobata* (a, b and c) and *Pocillopora* cf. *effusa* (d, e and f). From the Illumina sequences and assemblies, k-mer multiplicity was calculated. The x-axis is the k-mer multiplicity calculated from reads, and the numbers (0X, 1X, 2X, …) represent the k-mer multiplicity found in the corresponding assembly. K-mer multiplicity of 2 copies (blue curve) corresponds to duplicated contigs or overlaps in the assembly.

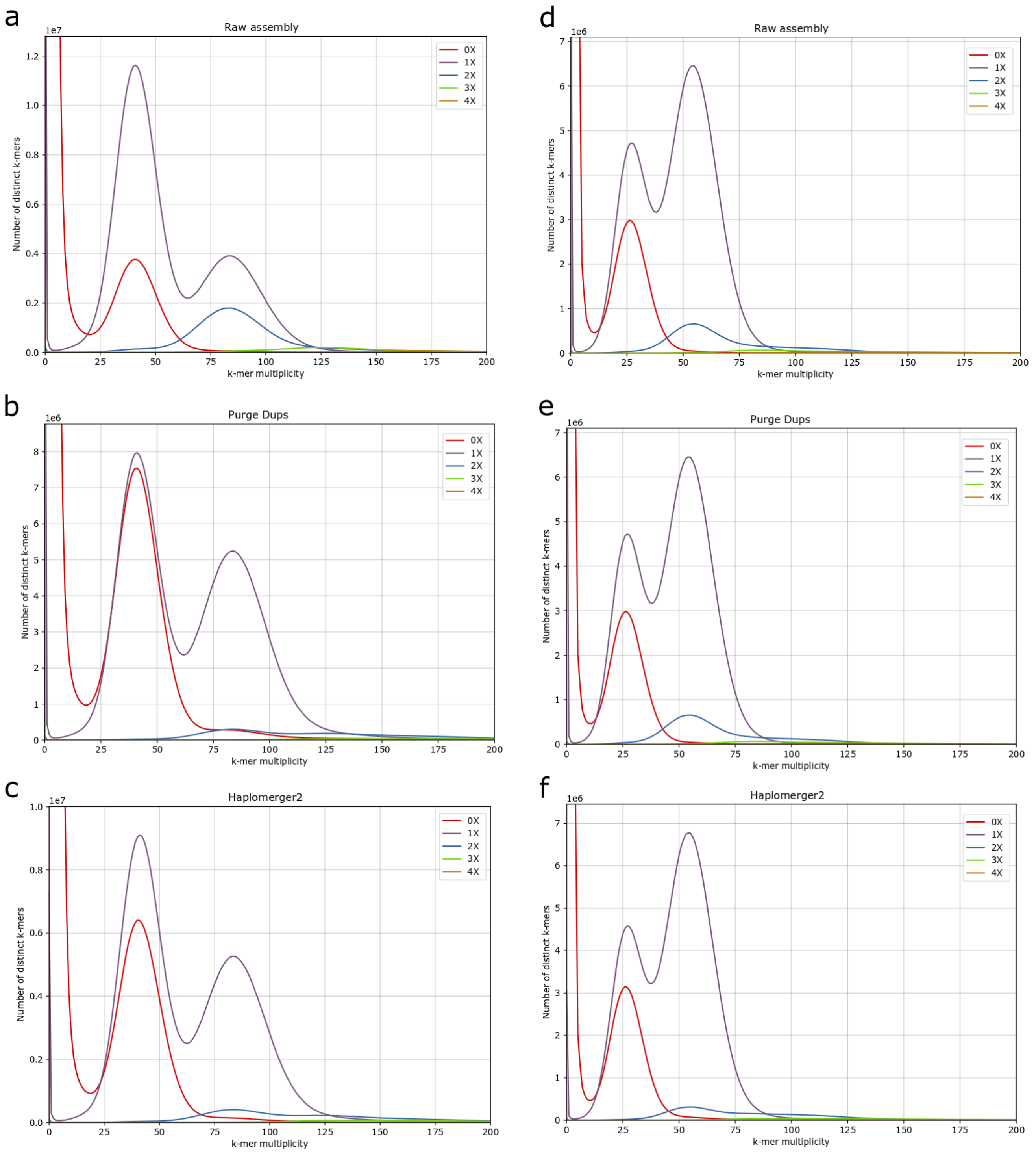

Figure S5. Distribution of the number of genes in tandemly repeated gene clusters for haplotype 1 and 2 assemblies generated by haplomerger2 for *Porites lobata* (A) and *Pocillopora* cf. *effusa* (B).

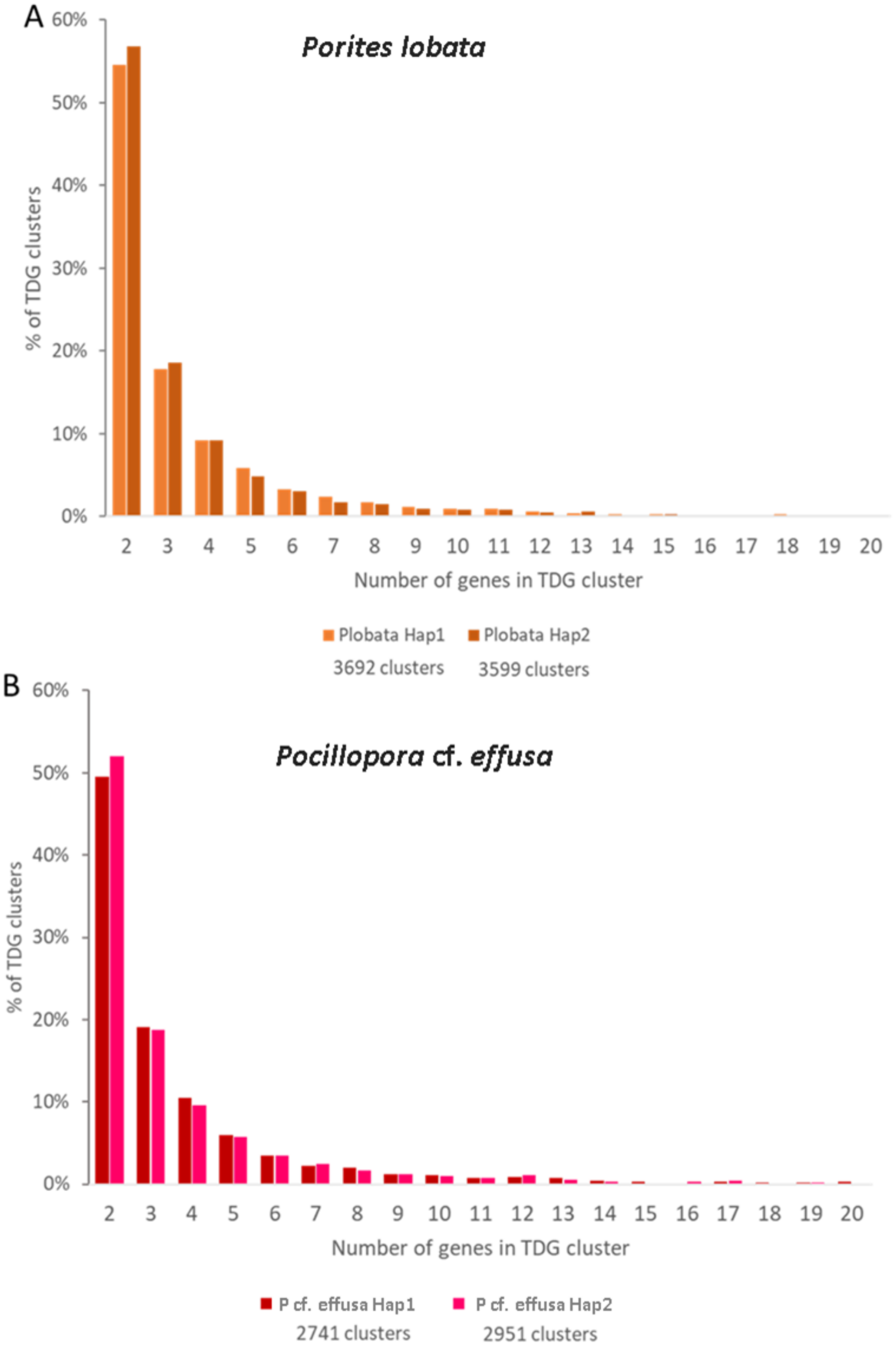

Figure S6. Validation of pairs of adjacent duplicated genes and clusters of TDG. Brown boxes represent three tandemly duplicated genes A, B and C, and blue rectangles represent ONT reads.

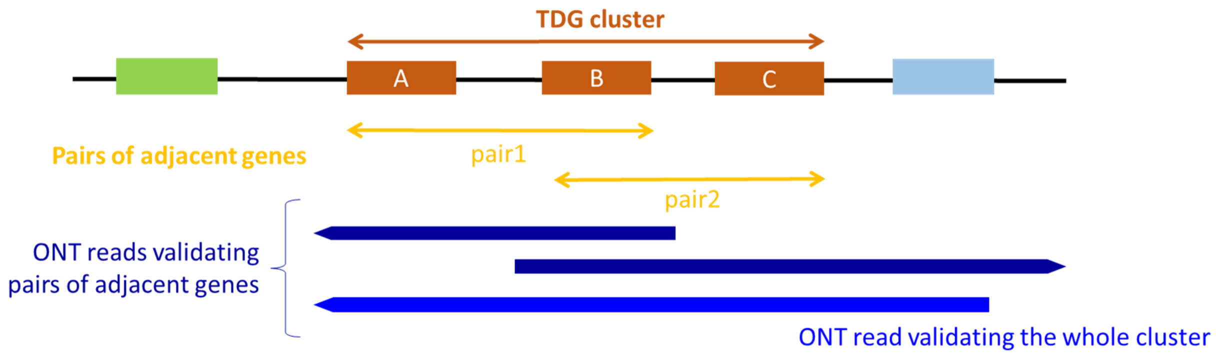

Figure S7. Validation of pairs of adjacent duplicated genes. Each dot represents a pair of two duplicated genes (**A**: *Porites lobata* N=8,220 and **B**: *Pocillopora* cf. *effusa* N=6,552) with x=0 for pairs that could not be validated by an ONT read and x=1 if at least one ONT read completely overlaps the two genes (Table S9). The y axis represents the distance from the start of the first gene to the end of the second gene. On the right panel, the read length distribution is given for comparison.

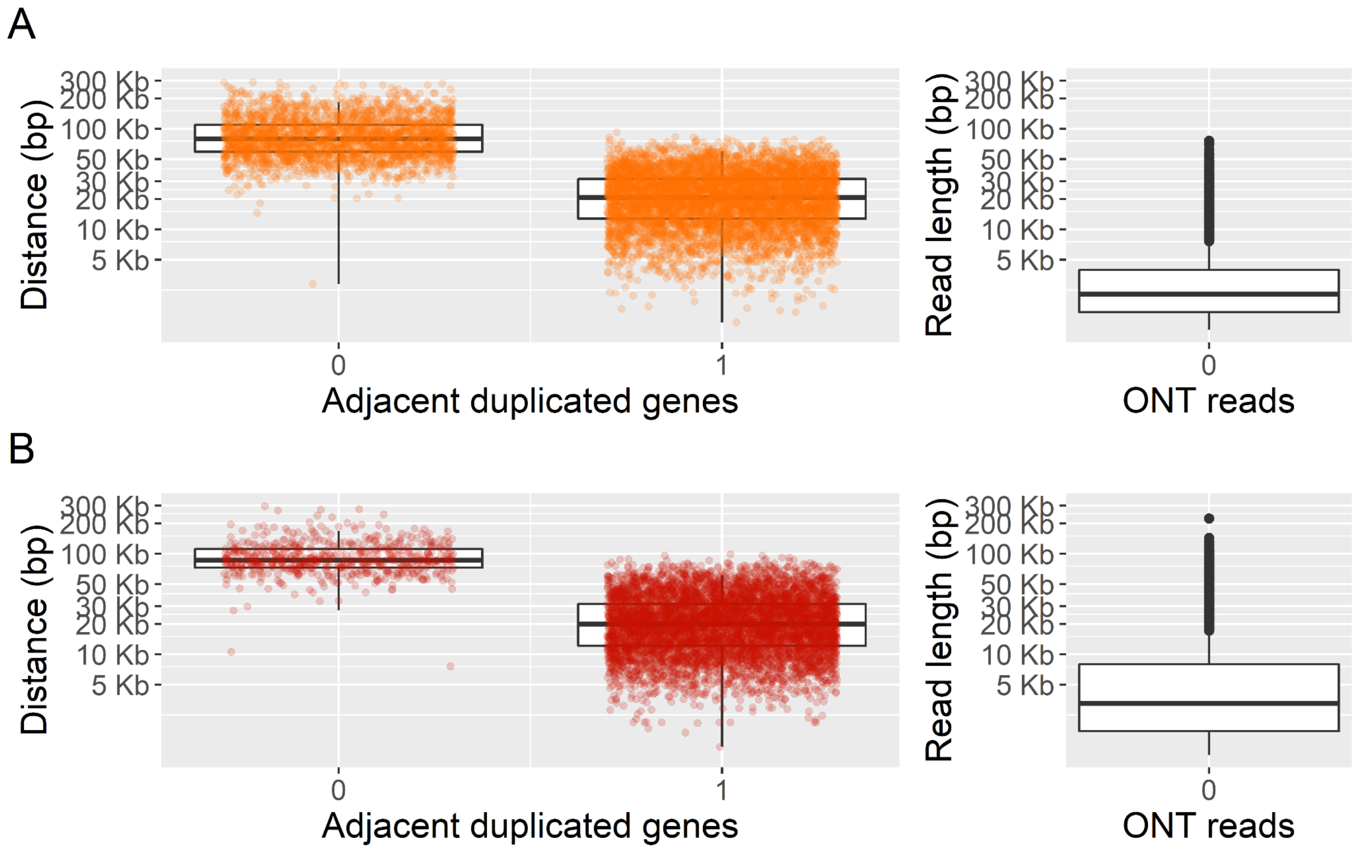

Figure S8. Validation of TDG clusters. Each dot represents a cluster of TDG (**A**: *Porites lobata* N=3,692 and **B**: *Pocillopora* cf. *effusa* N=2,741) with x=0 for clusters that could not be validated by an ONT read and x=1 if at least one ONT read completely overlaps all the genes of the cluster (Table S9). The y axis represents the distance from the start of the first gene to the end of the last gene. On the right panel, the read length distribution is given for comparison.

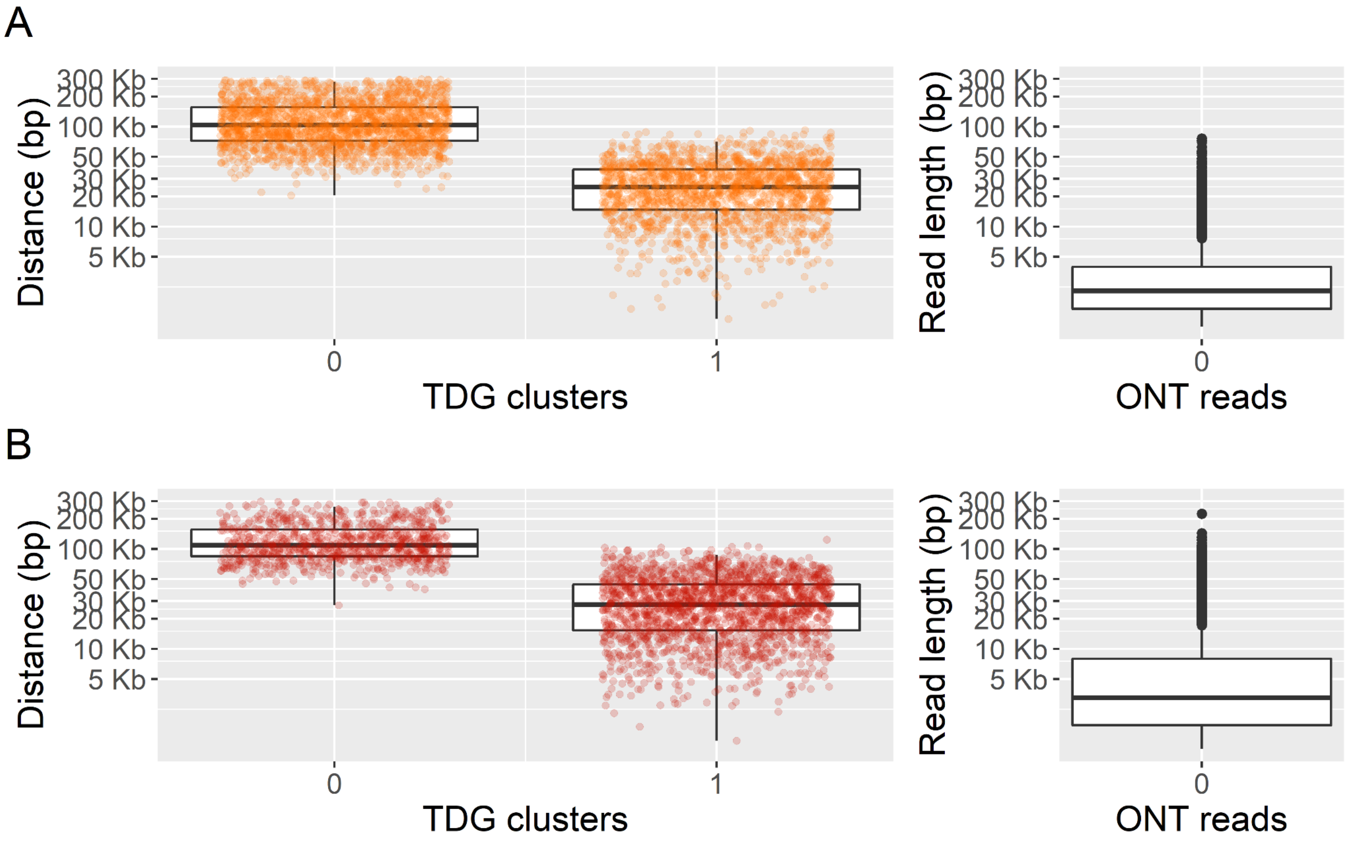

Figure S9. Comparison of haplotype assemblies and annotations for a TDG cluster (belonging to OG000308, amplified in corals, containing "GPCR" domain). **A**. Gbrowse view of Haplotype 1 (top) and 2 (bottom) assemblies. TDG cluster 3154 on haplotype 1 is validated by 10 nanopore reads spanning the whole cluster, and corresponds to TDG cluster 1055 on haplotype 2 (all gene pairs are BRH=best reciprocal hits). Ks between alleles are very low but some allelic differences are observed as seen in **B**. Zoom on an intronic region (gray triangle in A on Porites_lobata_contig_18:2,293,120..2,293,239) : allelic polymorphisms between haplotype 1 and 2 are validated by illumina and nanopore reads (one nanopore read is displayed for each haplotype). Reads are mapped on haplotype 1 and mismatches are displayed in yellow, deletions in red, insertions in purple.

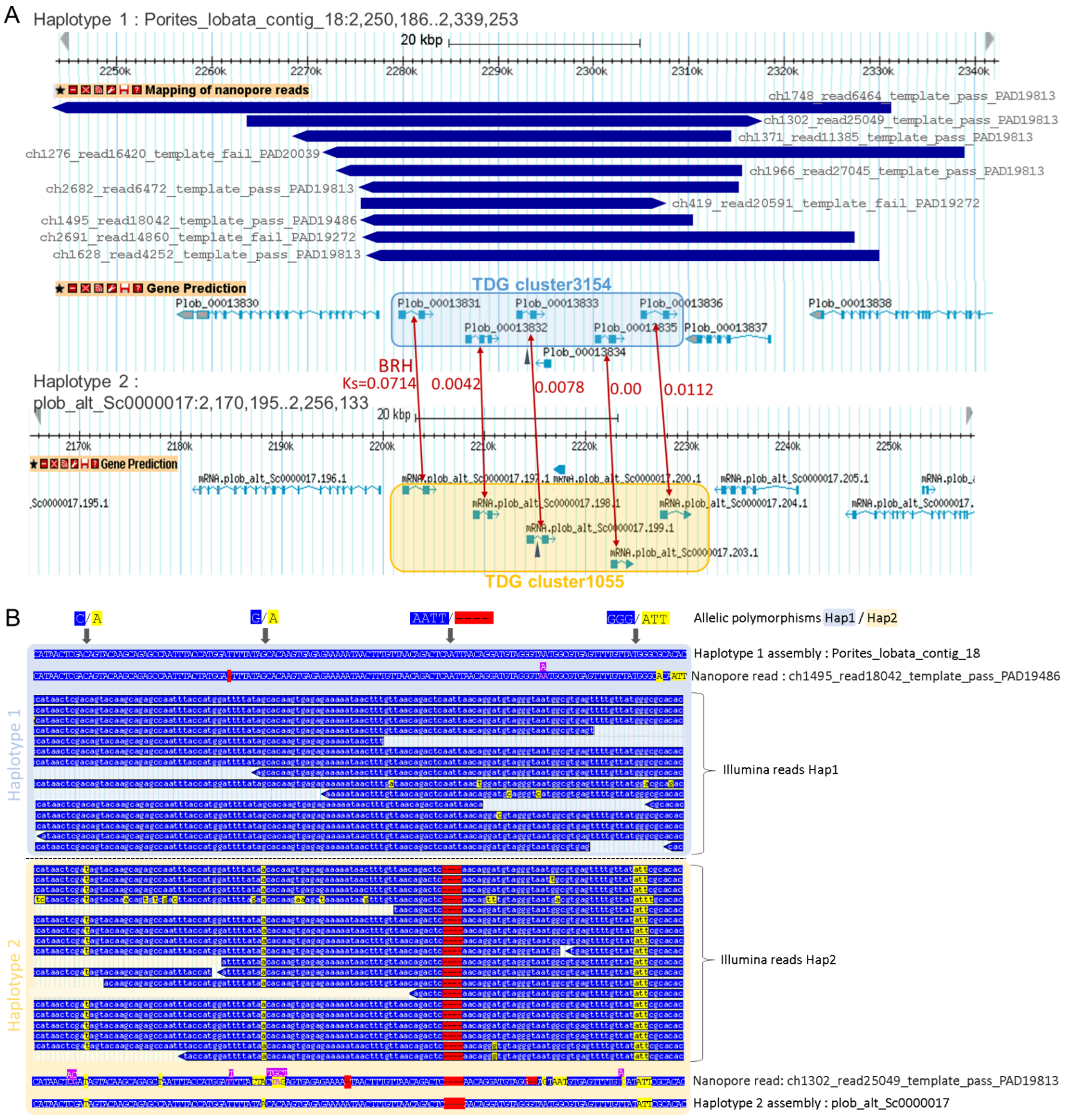

Figure S10. Two *P. lobata* genomic regions that illustrate proteins (A, green tracks) and transcripts (B, orange tracks) alignments before (middle track) or after (down track) the adaptation of gene prediction workflow for tandemly duplicated genes (“TDG patch”).

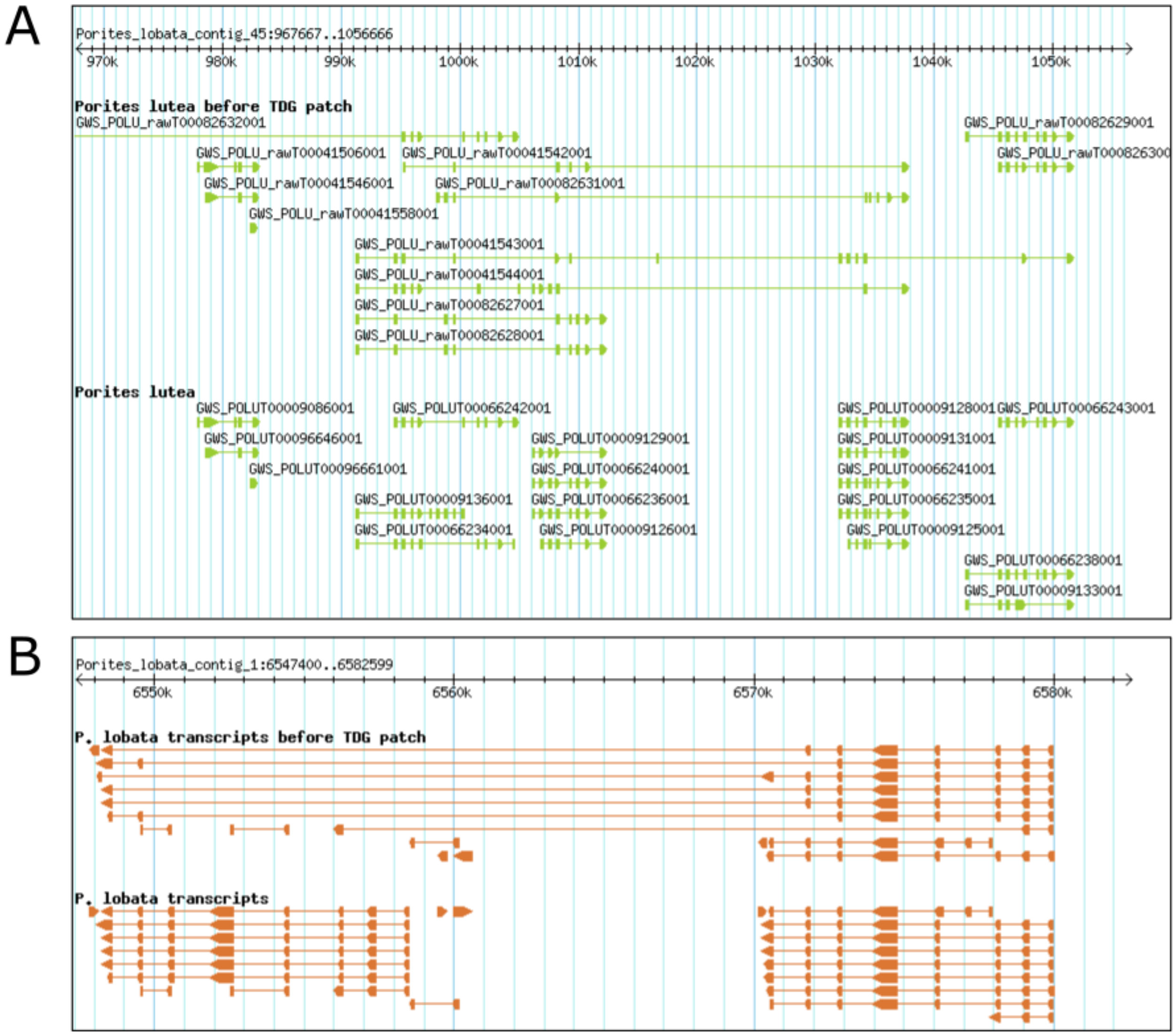

Figure S11. Consensus sequences of palindromic ITS satellites, found in *Porites lobata*, *Porites evermanni* and *Porites lutea* genomes. The telomeric palindrome is indicated by red arrows. These satellites are repeated in tandem in intergenic regions.

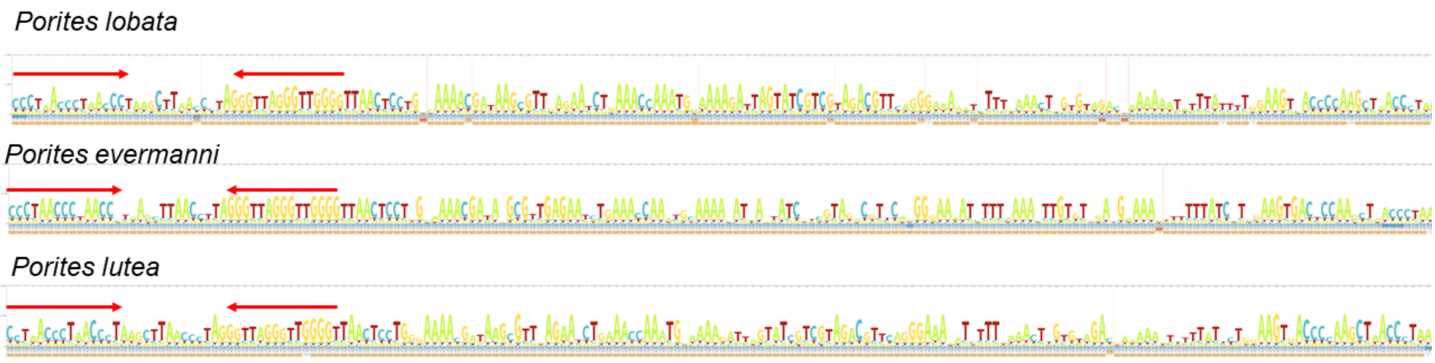

Figure S12. Conservation of orthologous genes in *Porites lobata* and *Pocillopora* cf. *effusa* with other Cnidarian. Distribution of identity percent between orthologs, grouped by clade.

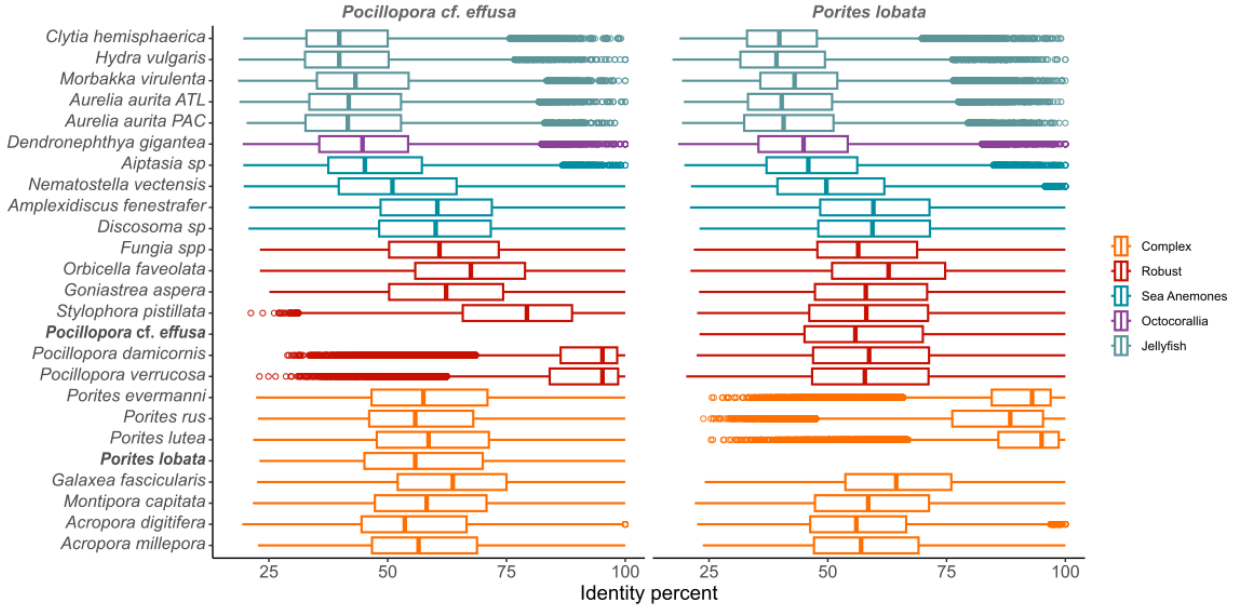

Figure S13. Number of TDG genes vs total number of genes on contigs. Total number of genes (x axis) vs number of TDG (with Ks<0.5) (y axis) for contigs with 10 genes or more in *Porites lobata (A)* and *Pocillopora* cf. *effusa* (B). Each dot represents a contig.

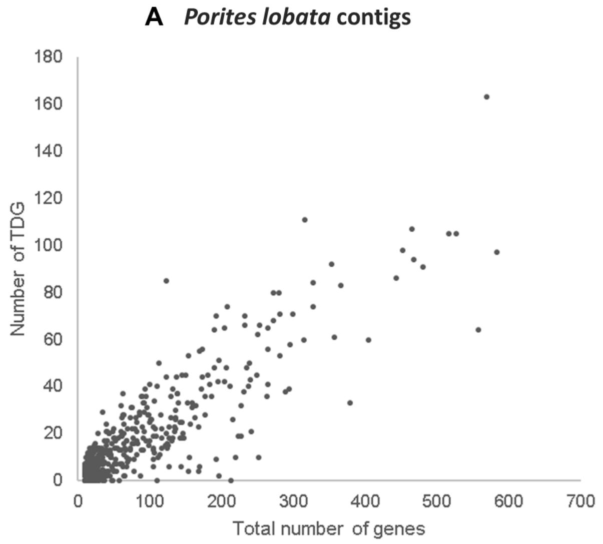

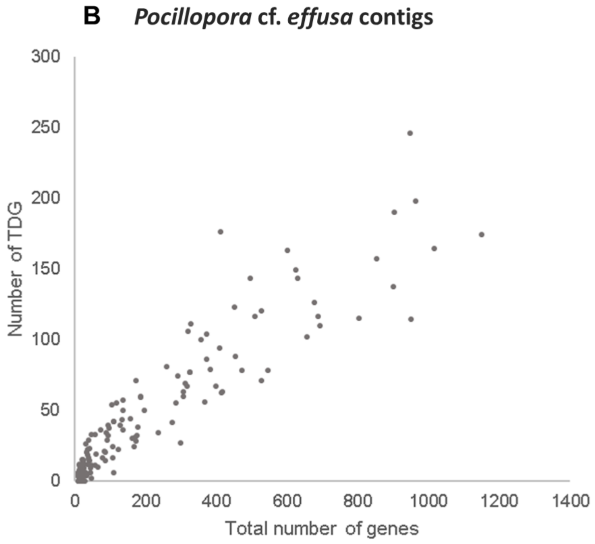

Figure S14. Expanded PFAM domains in corals vs non-corals hexacorallia.

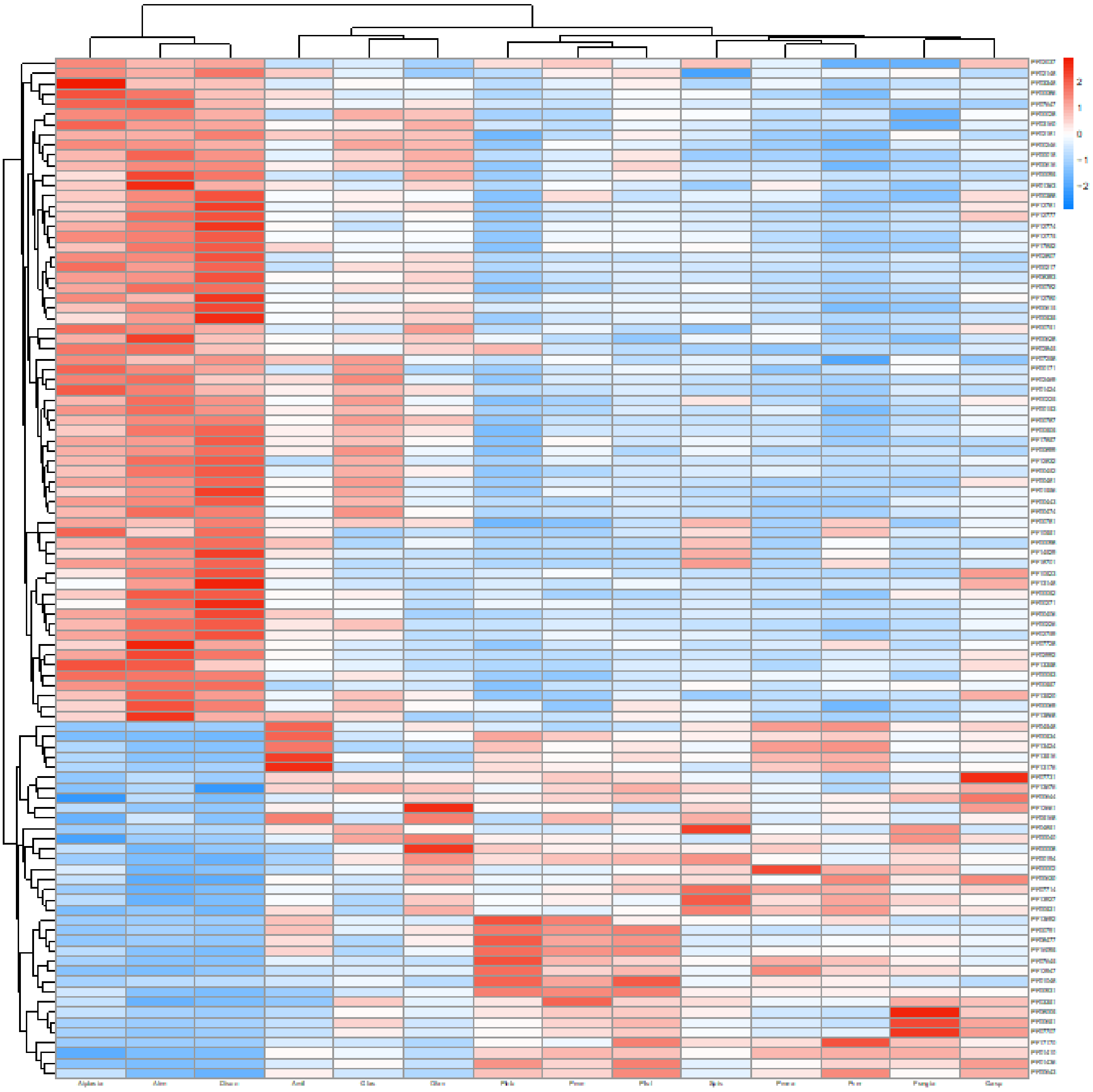

Figure S15. OGs amplified in complex vs robust corals. Heatmap representing the number of genes belonging to each OG.Complex and robust species are respectively grouped in the orange and dark red lines in the phylogenetic tree.

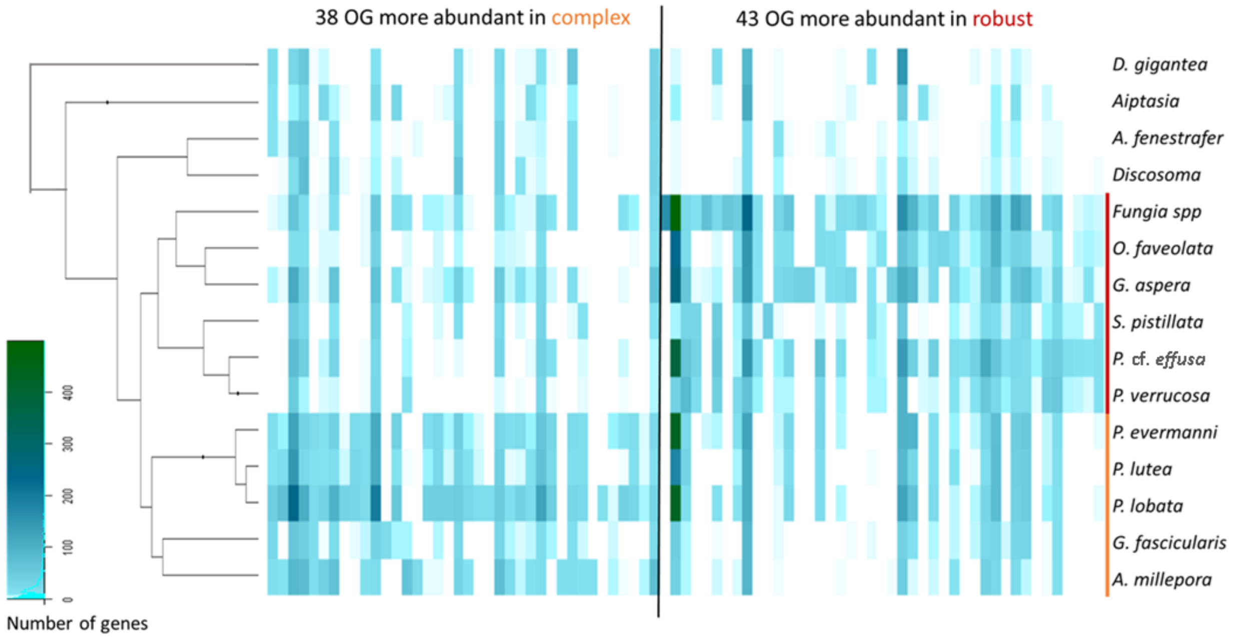

Figure S16. Expanded PFAM domains in complex vs robust corals.

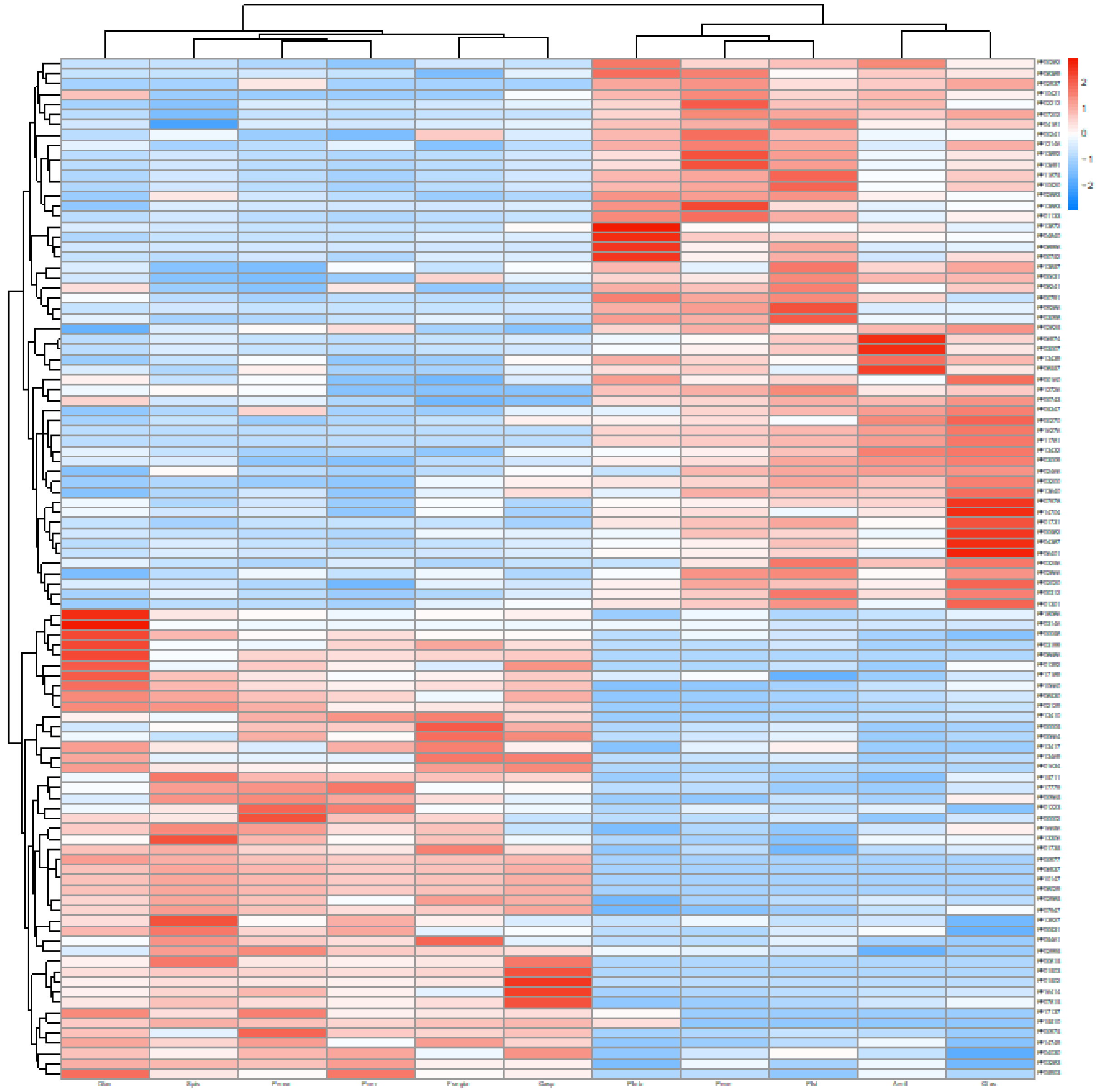

Figure S17. OGs amplified in massive vs branched corals. Heatmap representing the number of genes belonging to each OG. Green and purple lines show species belonging to massive and branched colonies (respectively). Species names are followed by C when belonging to the complex clade and R when belonging to the robust clade.

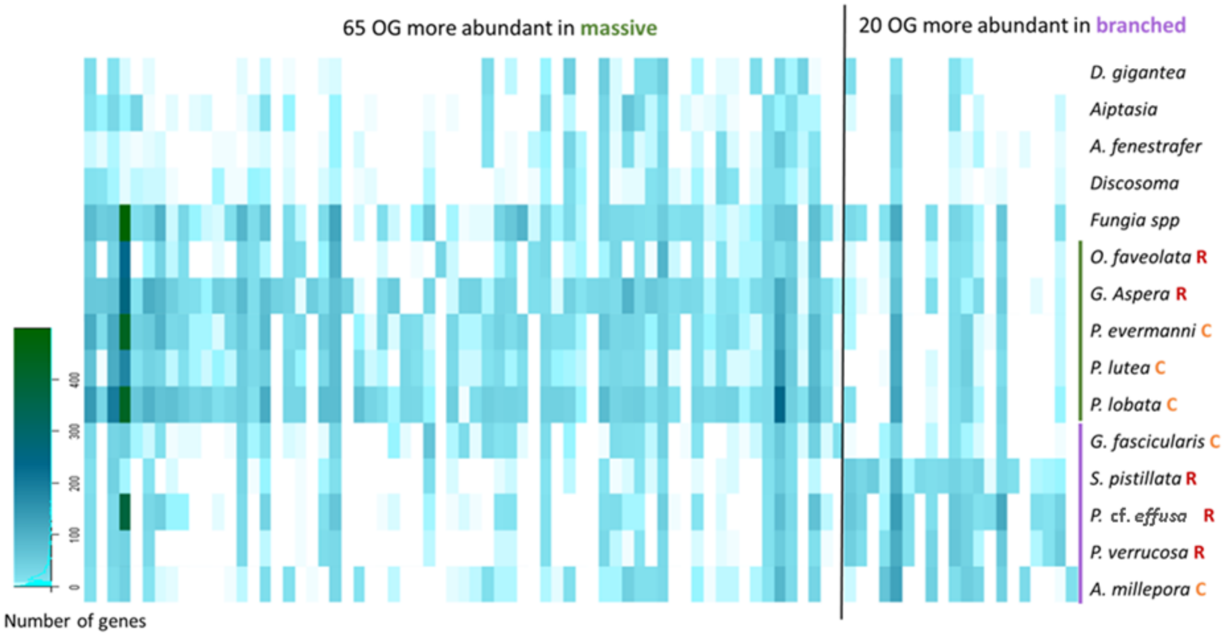

Figure S18. Heatmap representing the P-values obtained with CAFE for gene number amplification (in red) and reduction (in blue) events for each node on the phylogenetic tree. Species names are the following: neve= Nematostella vectensis, aisp= *Aispasia* sp, amfe= *Amplexidiscus fenestrafer*, disp= *Discosoma* sp, poru= *Porites rus*, poev= *Porites evermanni*, polo= *Porites lobata*, polu= *Porites lutea*, acmi= *Acropora millepora*, gafa= *Galaxea fascicularis*, orfa= *Orbicella faveolata*, goas= *Goniastrea aspera*, fusp= *Fungia* sp, poef= *Pocillopora* cf. *effusa*, poda= *Pocillopora damicornis*, pove= *Pocillopora verrucosa*, stpi= *Stylophora pistillata*.

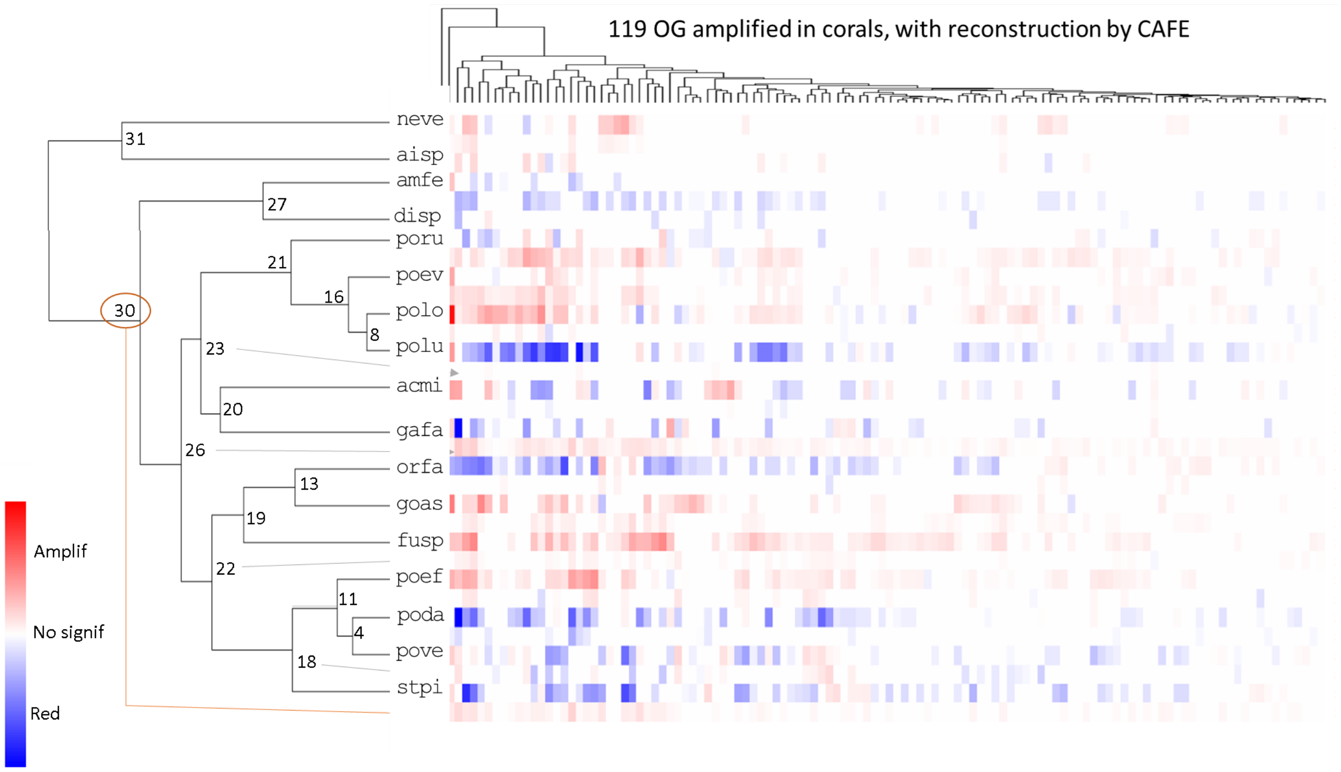

Figure S19. Gene family amplifications/reductions identified with CAFE. Results of CAFE reconstructions on gene families (orthogroups) present at the root of Hexacorallia (corals + sea anemones), in 17 species. Species names are the following: neve= Nematostella vectensis, aisp= *Aispasia* sp, amfe= *Amplexidiscus fenestrafer*, disp= *Discosoma* sp, poru= *Porites rus*, poev= *Porites evermanni*, polo= *Porites lobata*, polu= *Porites lutea*, acmi= *Acropora millepora*, gafa= *Galaxea fascicularis*, orfa= *Orbicella faveolata*, goas= *Goniastrea aspera*, fusp= *Fungia* sp, pome= *Pocillopora* cf. *effusa*, poda= *Pocillopora damicornis*, pove= *Pocillopora verrucosa*, stpi= *Stylophora pistillata*. **A.** phylogenetic tree constructed by orthofinder, with branches colored according to the number of significant gene family amplifications detected by CAFE, from white (0) to red (383, in the final branch leading to *Porites lobata*). **B.** Barplot representing the number of orthogroups (OG) with significant gene number variation for each species (all events that occured on the branches leading to the species are taken into account). The number of amplified gene families (OG) are in red and the number of contracted gene families (OG) are in blue.

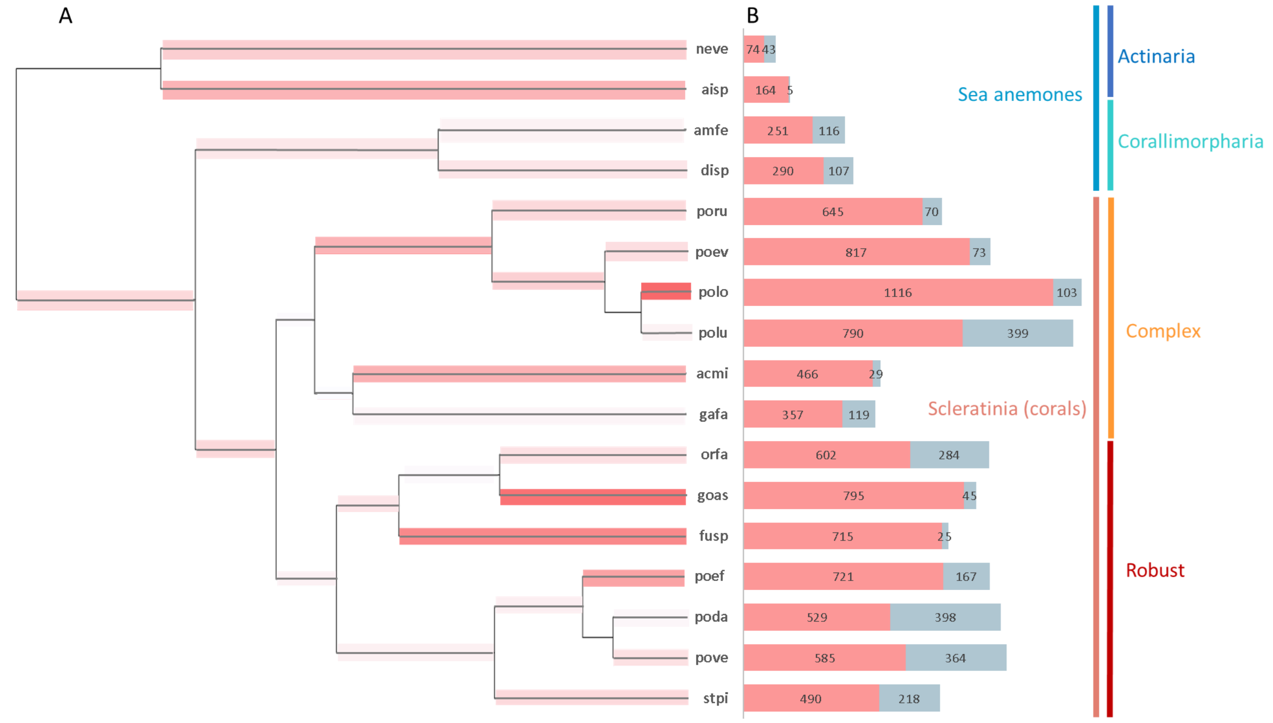

Figure S20. Distribution of Ks between TDG gene pairs in four classes of intergenic distances.

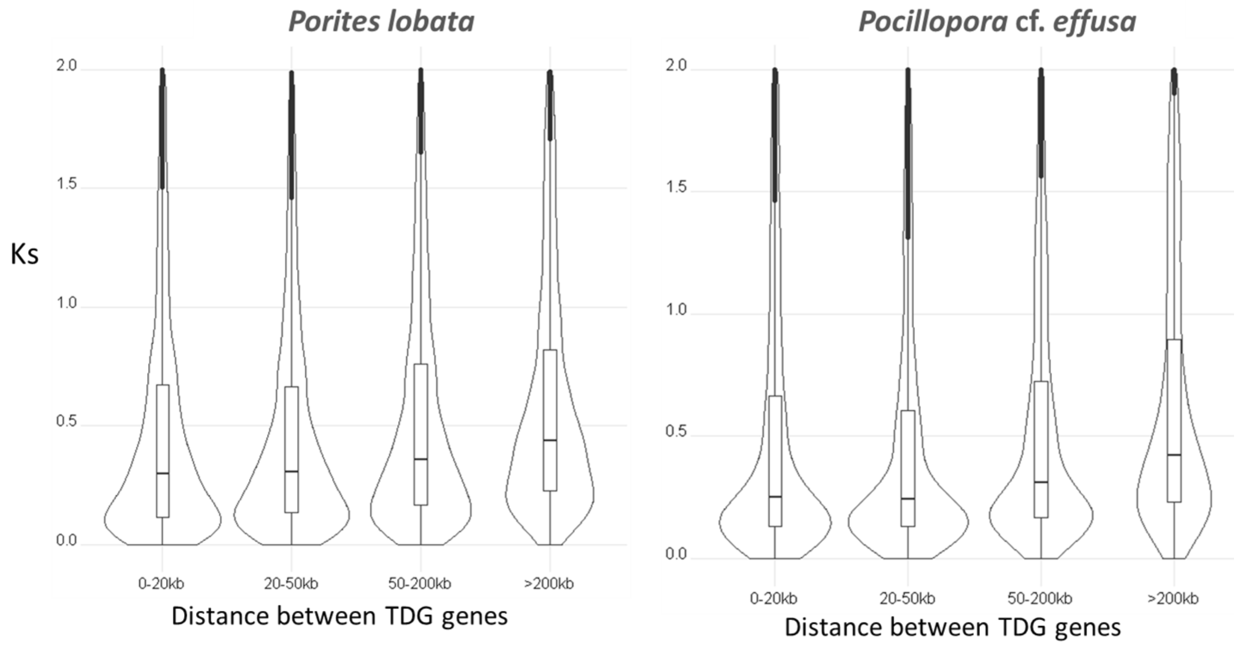

Figure S21. View of the *Pocillopora* cf. *effusa* genome browser, with 2 adjacent genes duplicated in tandem.

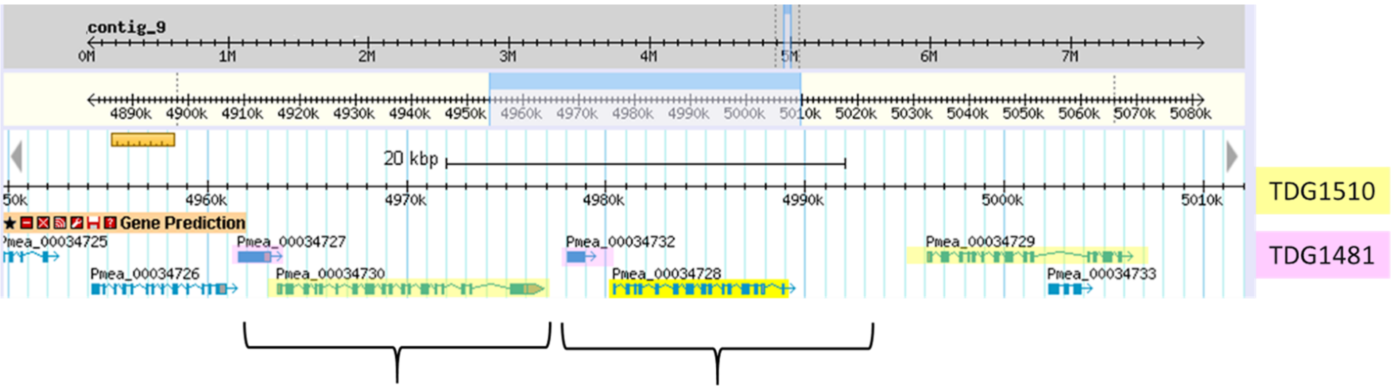

Figure S22. Distribution of Ks between TDG pairs in 3 structural conservation classes ("conserved" exons or introns is relative to the structure: it means that they have exactly the same size)**.** Numbers of pairs in each category are mentioned with "N=".

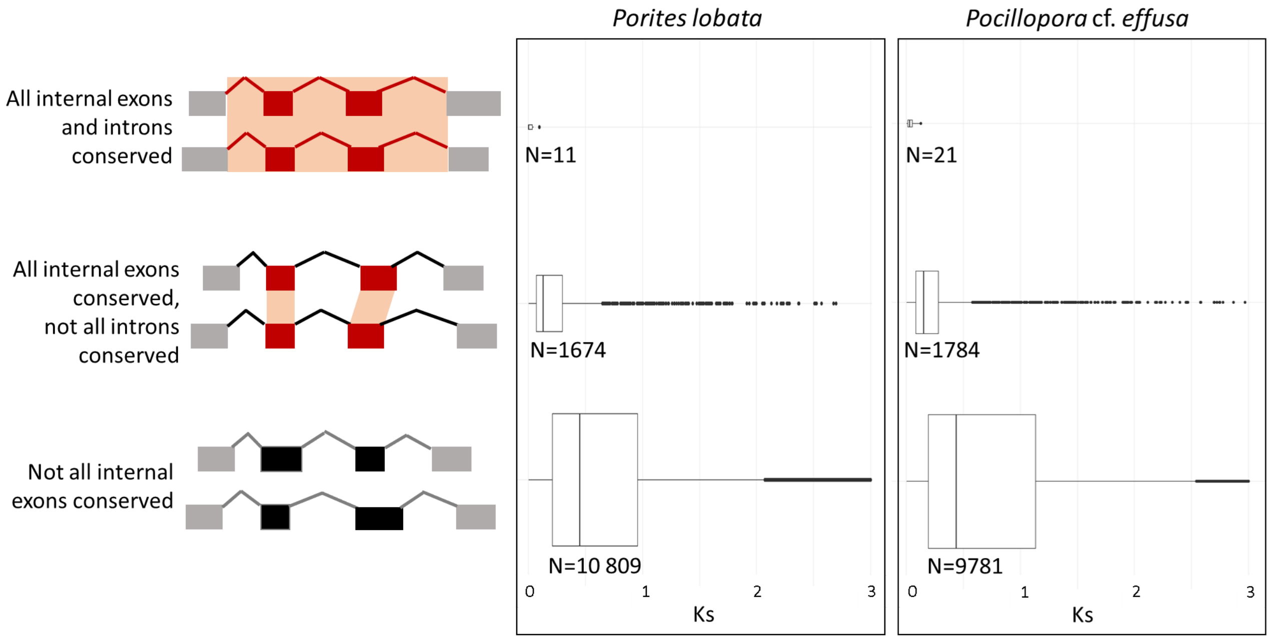

Figure S23. A. Browser view of a cluster of 3 tandemly duplicated genes belonging to OG0000023 (lectin-like domain). **B.** Structural comparison between the three annotated genes. **C.** Multiple alignment of 2 exons and one intron showing higher homology in exons.

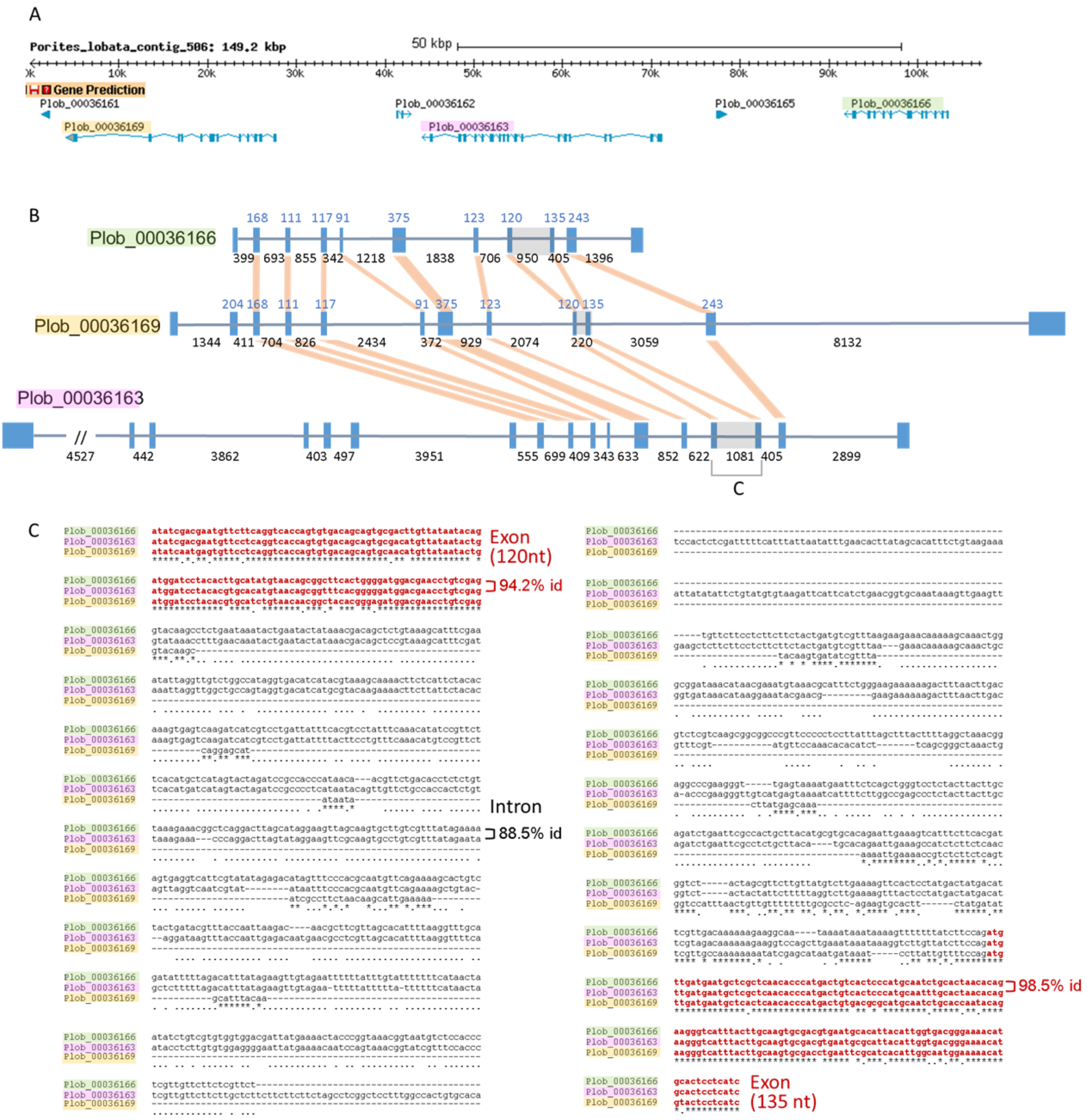

Figure S24. Expression quantification of 200 random OGs across the 103 meta-transcriptome samples. The heatmap shows the z-score of average TPM per OG (rows) according to the samples (columns). The z-score is computed by OG (row) using the scale function of the heatmap.2 from the gplots R package.

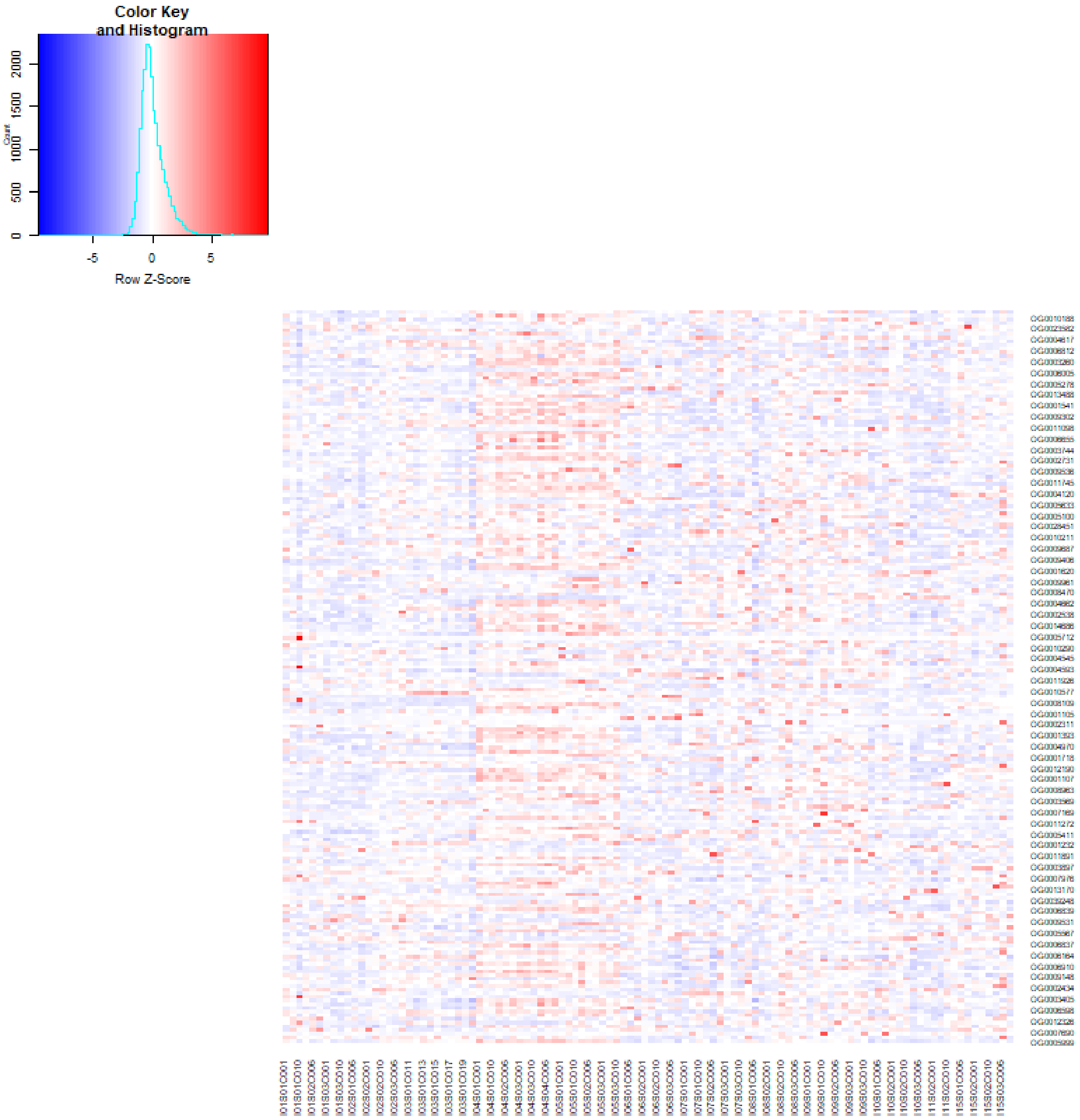

Figure S25. Number of genes in OG identified as TLR (A), NLR (B), lectins (C). Heatmaps (left panels) represent the number of genes in OGs for 14 cnidarian species. Histograms (right panels) represent the number of genes (TDG and not TDG) that belong to the annotated OG.

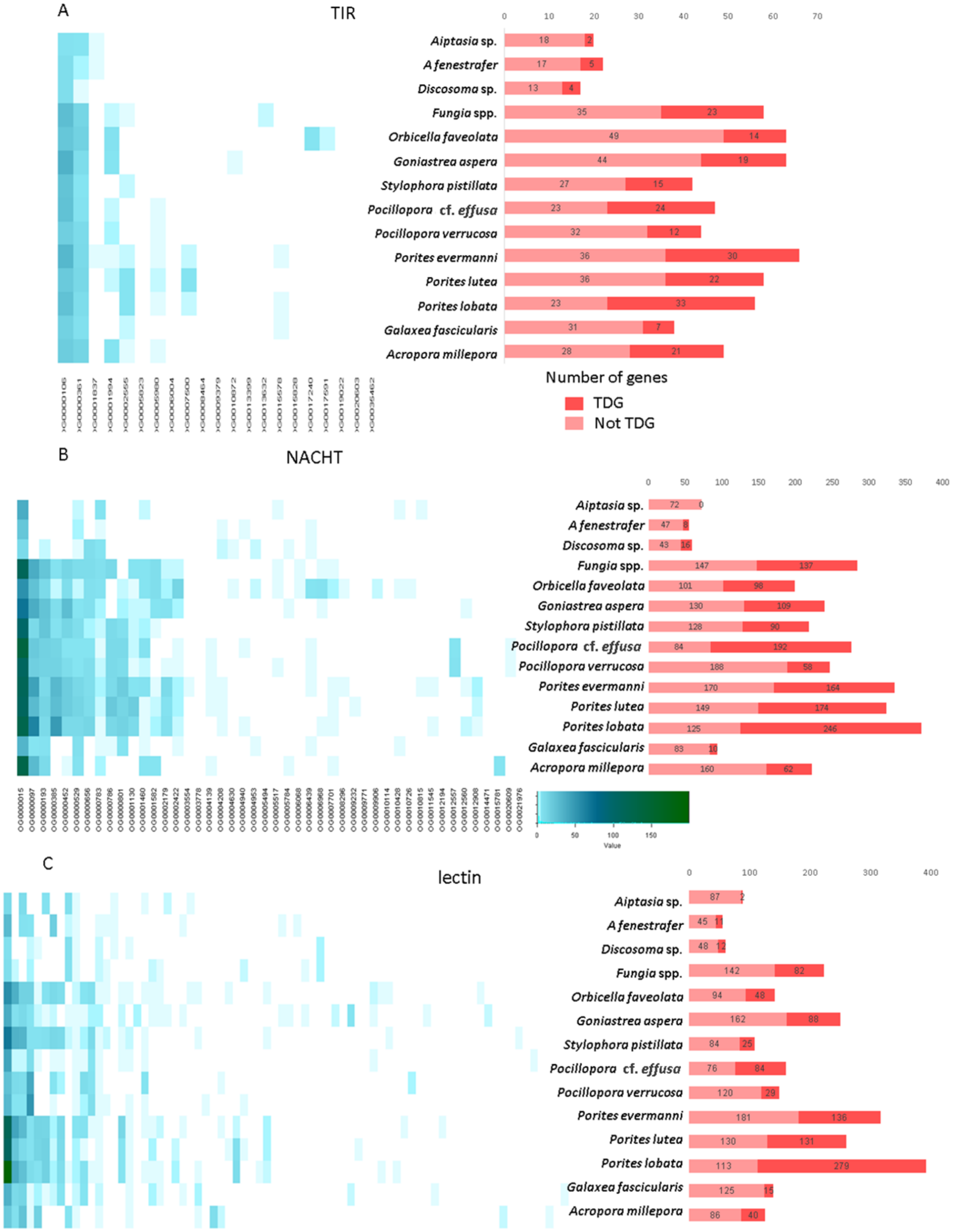

Figure S26. Percent of NACHT/NB-ARC containing proteins that are likely truncated at the N-terminal, C-terminal or both extremities (less than 250 aa upstream/downstream of the NACHT/NB-ARC domain), in 5 coral species. Total numbers of proteins are displayed with “N=”.

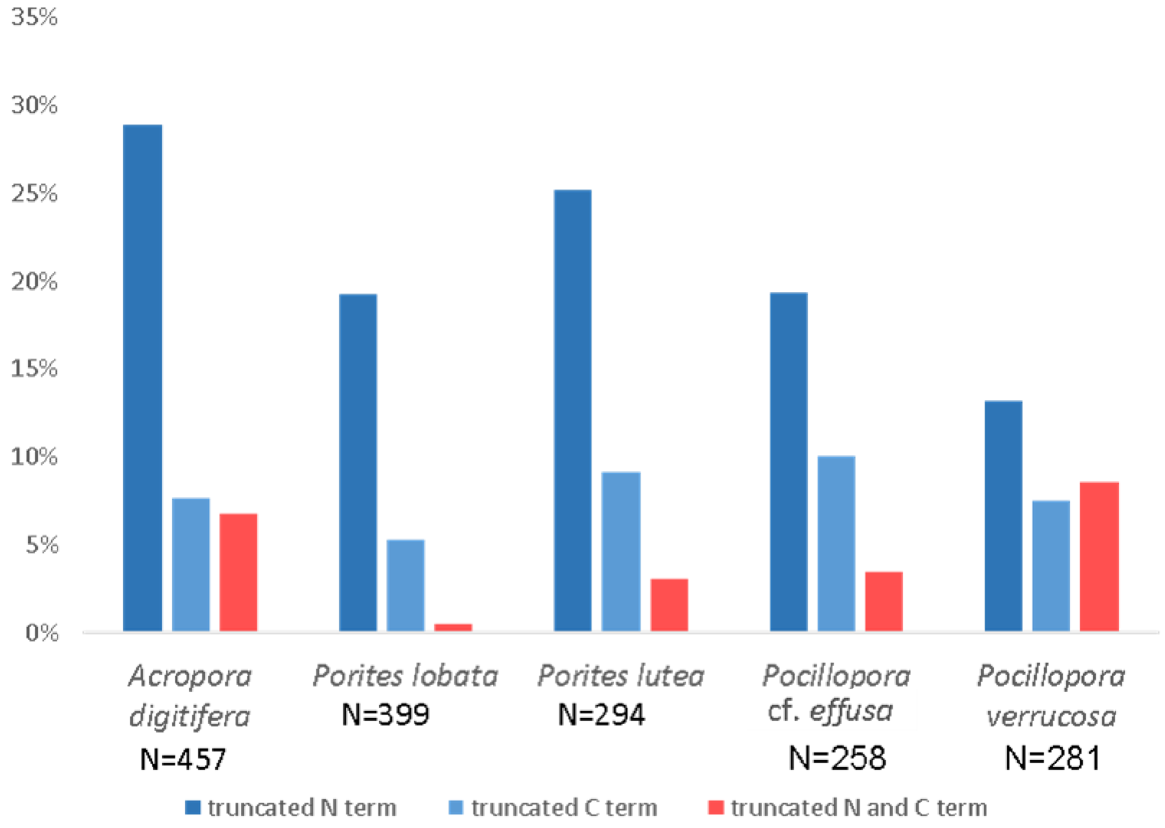

Figure S27. Observed N-terminal and C-terminal domains for NACHT/NB-ARC containing proteins in five coral species.

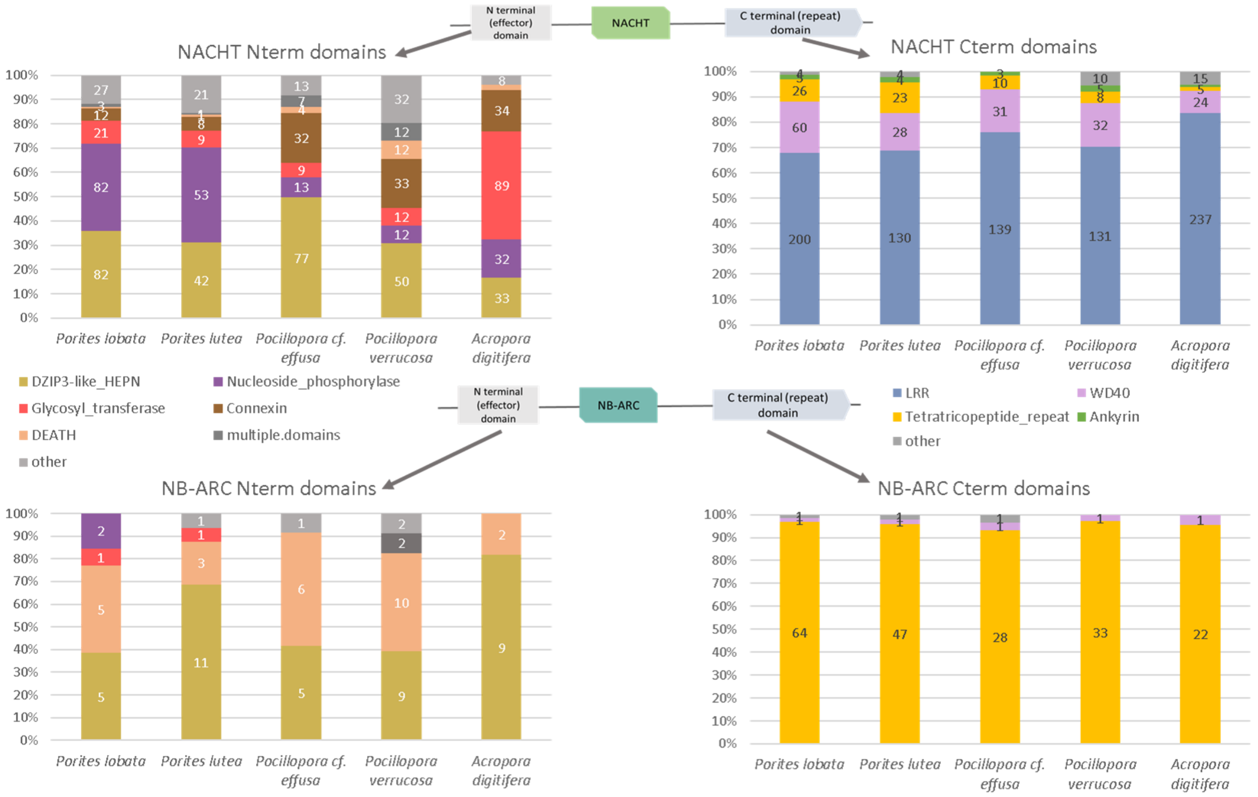

Figure S28. A. Graphic representation of the number of calcification-related proteins in non-scleractinian and scleractinian species **B.** *p*-values of calcification related proteins in non-scleractinian, scleractinian - robust and - complex coral species.

A.

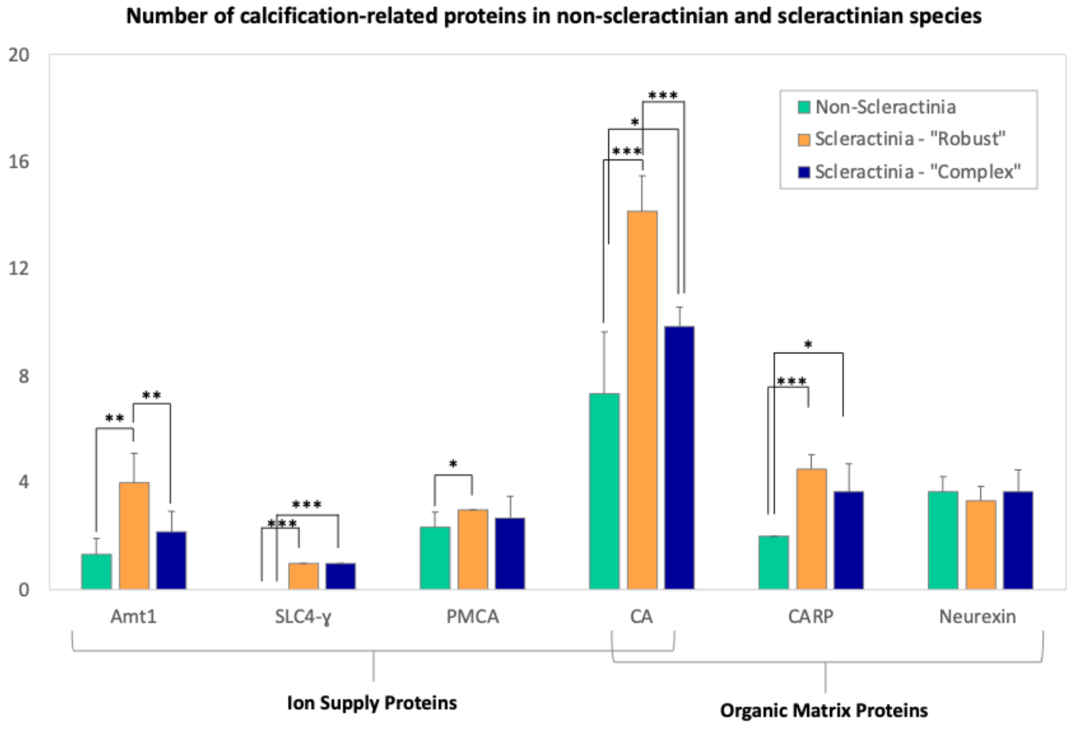

B.

p-value	Non-Scleractinia vs Scleractinia - Robust	Non-Scleractinia vs Scleractinia - Complex	Scleractinia - Robust vs Scleractinia - Complex
**Amt1**	0.00618 **	0.14	0.00702 **
**SLC4-γ**	<2e-16 ***	<2e-16 ***	0.341
**PMCA**	0.0185 *	0.553	0.341
**CA**	0.00067 ***	0.0383 *	0.0000395 ***
**CARP**	0.000122 ***	0.0306 *	0.111
**Neurexin**	0.407	1	0.418

Figure S29. Maximum likelihood phylogenetic tree (Phyml, LG + I + G) of cnidarian ammonium transporters Amt1. Cnidaria species include Corallimorpharia (*Discosoma sp.* - Dsp and *Amplexidiscus fenestrafer* - Afe), Actiniaria (*Exaiptasia diaphana* - Edi), Scleractinia - “Robust” (*Stylophora pistillata* - Spi, *Pocillopora verrucosa* - Pve, *Pocillopora* cf. *effusa* - Pme, *Orbicella faveolata* - Ofa, *Goniastrea aspera* - Gas, *Fungia sp.* - Fun) and Scleractinia - “Complex” (*Acropora digitifera* - Adi, *Acropora millepora* - Ami, *Galaxea fascicularis* - Gfa, *Montipora capitata* - Mca, *Porites lobata* - Plo, *Porites lutea* - Plu).

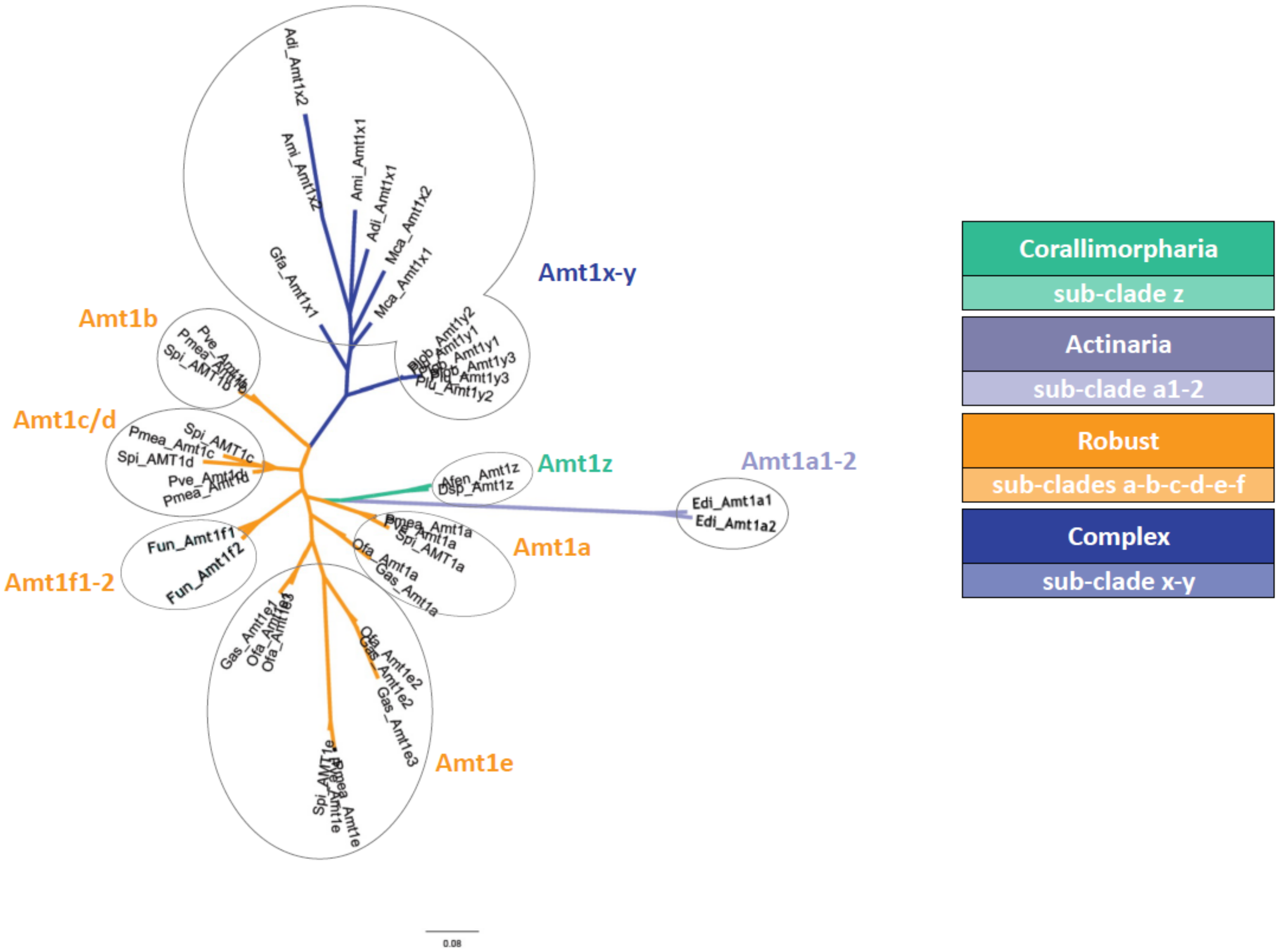

Figure S30. **Genomic localisation of cnidarian ammonium transporters Amt1 in various species**

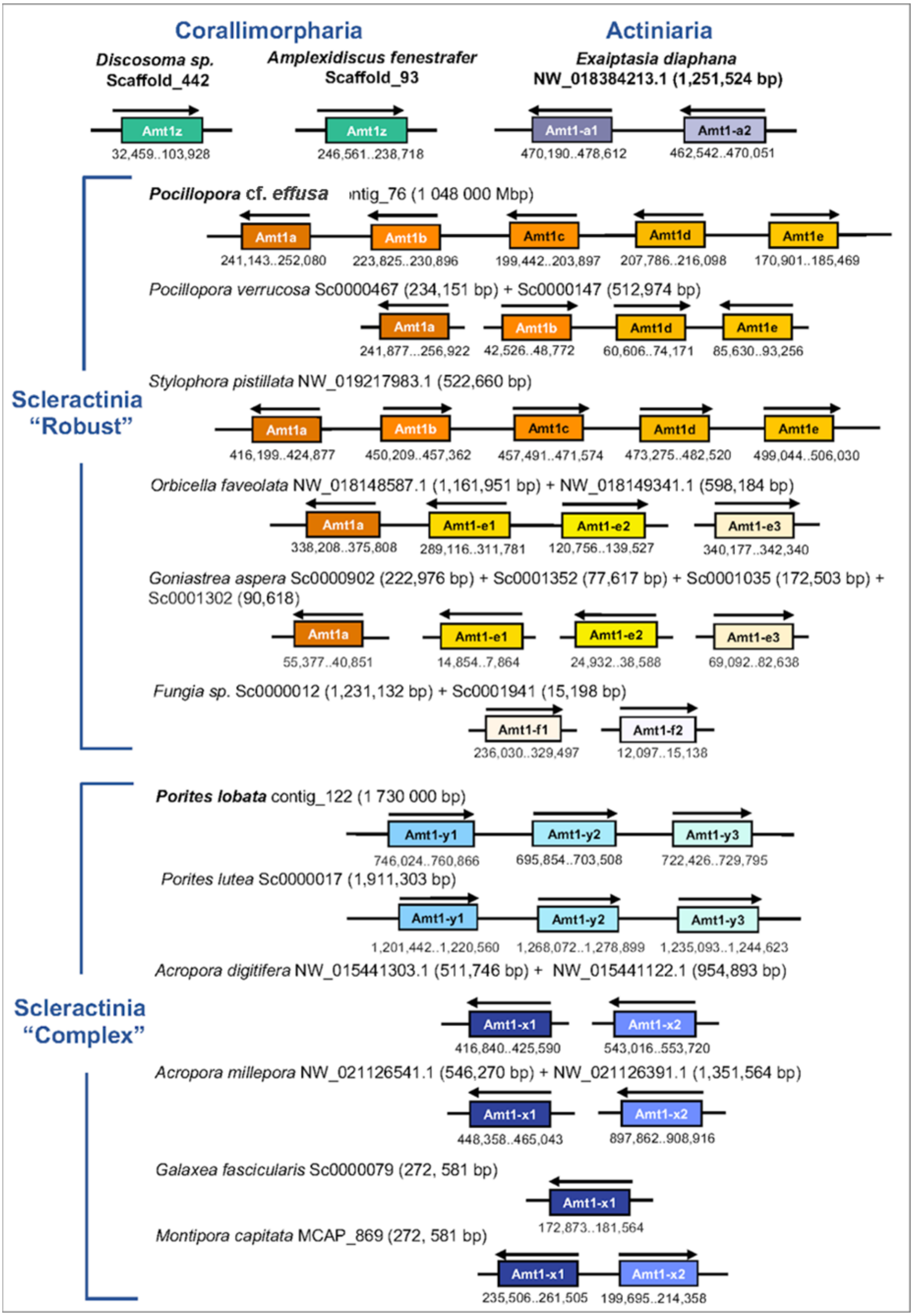

Figure S31. **Genomic localisation of Bicarbonate Anion Transporter SLC4 in various species.**

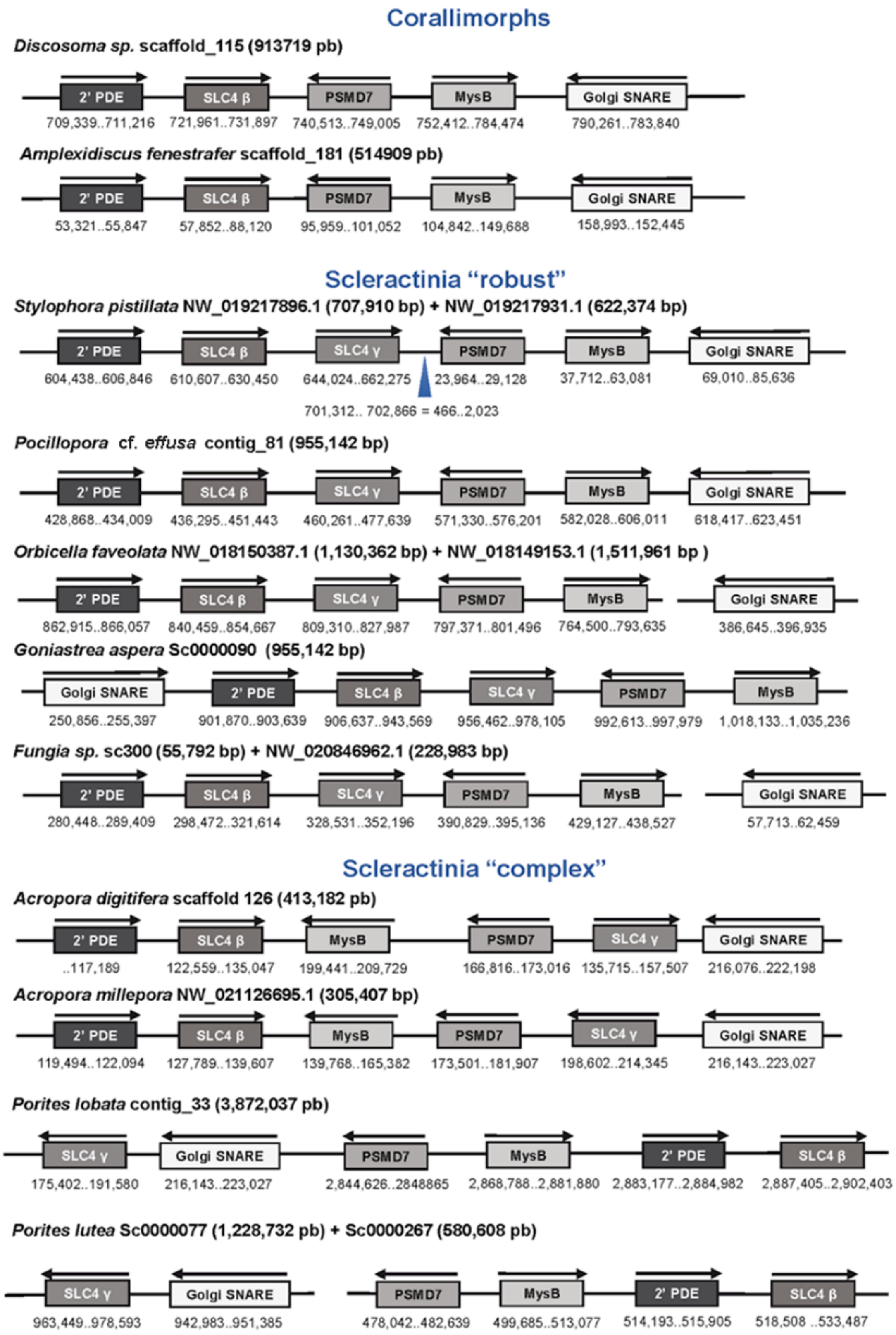

Figure S32. Coral monocopy gene set. Boxplots representing for each species the distribution of depth of mapping of short reads on 705 monocopy OG consensus (present in all coral species), normalized by the average depth on the 705 monocopy OGs consensus (A) or normalized by the average depth on 877 Busco metazoa ancestral sequences (B).

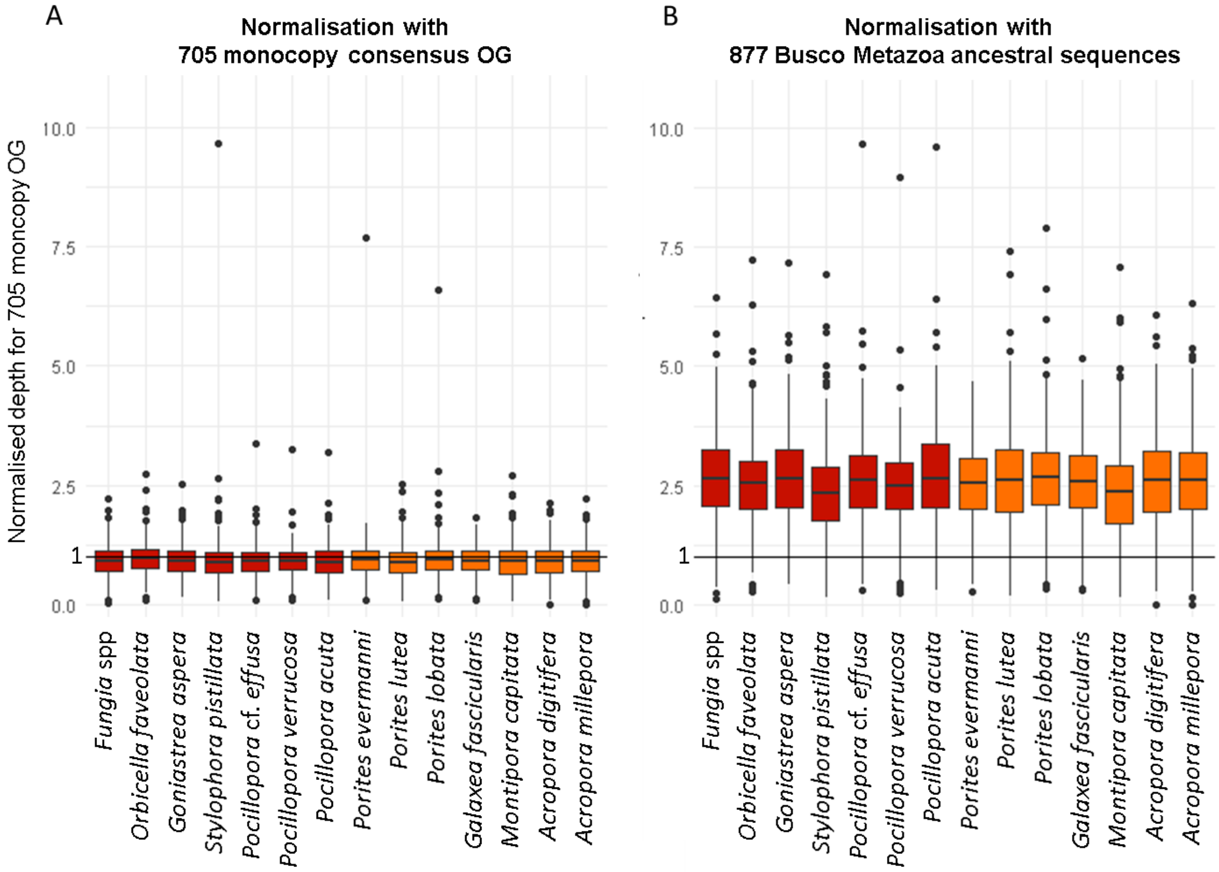

Supplement: Supplementary file 1 — Additional file 1: Table S1. Statistics of the ONT sequencing data. Table S2. Raw long-read assemblies. A. Porites lobata. B. Pocillopora cf. effusa. Table S3. Statistics of all assemblies. Table S4. Statistics of gene prediction on reference and alternative haplotype assemblies. Table S5. BUSCO analyses of raw and haploid assemblies. Table S6. Repeat composition of coral assemblies. Table S7. Data used for comparison of 26 Cnidaria species. Table S8. Coverage of genome assemblies by syntenic blocks (for contigs with at least 5 genes). Table S9. Validation of pairs of adjacent duplicate genes and clusters of tandemly duplicated genes. Table S10. Table of calcification-related proteins in non-scleractinian and scleractinian - robust and - complex coral species. Figure S1. K-mer distribution and estimation of genome sizes and heterozygous rates from short reads. Figure S2. KAT plot of Porites lobata assemblies. Figure S3. KAT plot of Pocillopora cf. effusa assemblies. Figure S4. K-mer profiling for all assemblies of Porites lobata and Pocillopora cf. effusa Figure S5. Distribution of the number of genes in tandemly repeated gene clusters. Figure S6. Validation of pairs of adjacent duplicated genes and clusters of TDG. Figure S7. Validation of pairs of adjacent duplicated genes. Figure S8. Validation of TDG clusters. Figure S9. Comparison of haplotype assemblies and annotations for a TDG cluster. Figure S10. Two P. lobata genomic regions that illustrate proteins and transcripts alignments before or after the adaptation of gene prediction workflow for tandemly duplicated genes. Figure S11. Consensus sequences of palindromic ITS satellites, found in Porites lobata, Porites evermanni and Porites lutea genomes. Figure S12. Conservation of orthologous genes in Porites lobata and Pocillopora cf. effusa with other Cnidarian. Figure S13. Number of TDG genes vs total number of genes on contigs. Figure S14. Expanded PFAM domains in corals vs non-corals hexacorallia. Figure S15. [file 13059_2023_2960_MOESM1_ESM.pdf]
